# Amyloid spatial extent with florbetapir-PET for early detection of preclinical Alzheimer’s disease

**DOI:** 10.1016/j.tjpad.2026.100529

**Published:** 2026-03-13

**Authors:** Emma G. Thibault, Grace Del Carmen Montenegro, J․Alex Becker, Julie C․ Price, Brian C. Healy, Bernard J. Hanseeuw, Rachel F. Buckley, Heidi I.L. Jacobs, Michael J. Properzi, Reisa A. Sperling, Keith A. Johnson, Michelle E. Farrell

**Affiliations:** a Department of Radiology, Massachusetts General Hospital, Harvard Medical School, 55 Fruit St, Boston, MA, 02114, USA; b Department of Neurology, Massachusetts General Hospital, Harvard Medical School, 55 Fruit St, Boston, MA, 02114 USA; c Biostatistics Center, Massachusetts General Hospital, Harvard Medical School, 55 Fruit St Boston, MA, 02114, USA; d Department of Neurology, Cliniques Universitaires Saint-Luc, Université Catholique de Louvain, *Av*. Hippocrate 10, 1200 Bruxelles, Belgium; e Melbourne School of Psychological Sciences, University of Melbourne, Melbourne VIC 3052, Australia; f CenterCenter for Alzheimer Research and Treatment, Department of Neurology, Brigham and Women’s Hospital, Harvard Medical School, 221 Longwood Avenue Boston, MA, 02115, USA

**Keywords:** Amyloid, PET, Preclinical Alzheimer’s disease, pTau217, Early detection, Amyloid staging

## Abstract

**Background:**

Prevention of Alzheimer’s disease (AD) requires biomarkers sensitive to the earliest amyloid**-**β (Aβ) deposits**.**

**Objectives:**

To characterize performance of a recently-developed Aβ-PET spatial extent metric (EXT) for early Aβ detection using ^18^[F]-florbetapir (FBP)-PET, evaluating its sensitivity, reliability, and associations with plasma pTau217, tau-PET, and cognition**.**

**Design:**

Longitudinal study with up to 5.5 years of PET, plasma and cognitive measures.

**Setting:**

The Anti-Amyloid Treatment in Asymptomatic Alzheimer’s disease (A4) Study and its companion screen-fail study Longitudinal Evaluation of Amyloid and Neurodegeneration Risk (LEARN) conducted across 67 international sites.

**Participants:**

1118 cognitively unimpaired older adults from the A4 placebo arm and LEARN.

**Measurements:**

EXT (% of neocortex above region-specific thresholds), global Aβ SUVR, plasma pTau217, medial temporal (MTL) and temporal neocortical (nTEMP) tau-PET SUVR, and Preclinical Alzheimer Cognitive Composite (PACC).

**Results:**

EXT showed high cross-sectional reliability and longitudinal stability. Using EXT reclassified 21.4% of SUVR-participants from Aβ− to Aβ+ and predicted who would progress to SUVR+ 5.5 years later with 83% sensitivity and 94% specificity. In SUVR− individuals, higher pTau217 associated with greater SUVR only within EXT+ individuals. Baseline EXT outperformed SUVR in predicting MTL tau proliferation. For neocortical tau SUVR and PACC change, EXT was the better predictor in earlier Aβ stages (while Aβ spread) while SUVR was superior later (after Aβ was widespread).

**Conclusions:**

EXT is a robust, generalizable PET metric that detects Aβ before global positivity and early Aβ-related changes in tau and cognition, supporting its relevance for trial enrichment and early therapeutic monitoring in AD prevention trials.

## Highlights

• Florbetapir spatial extent replicates PiB findings in preclinical AD.
• Spatial extent detects early amyloid-β spread below SUVR thresholds.
• EXT predicts early medial temporal tau accumulation and plasma pTau217 levels.
• Spatial extent improves early detection and could improve trial enrichment in AD.

## 1 Introduction

Alzheimer’s disease (AD) research is increasingly focused on earlier stages of the disease trajectory because recent successful anti-amyloid beta (Aβ) clinical trials in those with prodromal or mild AD [[1], [2], [3], [4], [5]] provide evidence that Aβ removal had greater clinical benefit in those in earlier stages of tauopathy [2,6]. Biomarkers optimized to detect the earliest pathological changes are therefore needed.

We recently reported the development of a novel metric for Aβ-PET that estimates the spatial extent (EXT) of Aβ deposition by calculating the proportion of Aβ-vulnerable neocortex in which elevated Aβ signal is detected [7]. In a cohort of cognitively unimpaired older adults from the Harvard Aging Brain Study (HABS), we demonstrated that Aβ EXT was sensitive to the early, focal Aβ deposits that fell below a conventional global Aβ distribution volume ratio (DVR) threshold for positivity, reclassifying an additional 11.2 % of the sample from Aβ- to Aβ+ based on EXT. Simply lowering the global Aβ threshold introduced false positives that were correctly avoided with EXT, confirmed at longitudinal PET follow-up. Beyond this advantage for early detection, EXT also provided a continuous measure of how far Aβ has spread that was more strongly associated with future tau proliferation and cognitive decline than conventional global Aβ level metrics. Using baseline and longitudinal data, we evaluated three stages of amyloid extent: EXT0 where EXT is too low to differentiate signal from noise, EXT1 where Aβ is spreading, and EXT2 where Aβ is widespread and detectable in all or nearly all Aβ-vulnerable neocortex. We observed early changes in tau pathology and cognitive decline among the EXT1 participants, which suggested that this stage may provide a suitable window for trials targeting the earliest detectable progression. Furthermore, while cognitive decline and tau pathology continued to worsen in the EXT2 stage, these changes did not associate with further increases in Aβ DVR, suggesting it is Aβ EXT rather than concentration that is more strongly linked to downstream associations with tau and cognition.

While our previous findings provided proof-of-concept that this Aβ EXT approach provides meaningful information about amyloidosis, replication in an independent sample is essential to confirm that the method and its advantages during preclinical AD are generalizable. While the ^11^C-Pittsburgh Compound B (PiB) Aβ tracer and single PET system in HABS were ideal for the initial development of our Aβ EXT approach, it is not yet clear if EXT will remain a sensitive Aβ metric when employing historically more accessible ^18^F-tracers with reduced gray-to-white matter contrast typical of multi-site AD clinical trials. The additional sources of noise, particularly high non-specific binding in white matter [[8], [9], [10]], may obscure the definition of robust positivity thresholds [11,12]. This issue is compounded for EXT due to its reliance on 42 ROI-level thresholds for regional Aβ+ rather than a single global Aβ+ threshold, raising concerns that the EXT approach may be inaccurate or unreliable for broader use.

The present study seeks to replicate our previous work [7] using [^18^F]-florbetapir-PET data from cognitively normal individuals in the placebo group of the Anti-Amyloid Treatment in Asymptomatic Alzheimer’s disease (A4) clinical trial, and its companion study Longitudinal Evaluation of Amyloid and Neurodegeneration Risk (LEARN). In this study, we examine the association between EXT and global neocortical Aβ (standardized uptake volume ratio, SUVR), cross-sectional and longitudinal stability of EXT with FBP, and its ability to improve early Aβ detection and prediction of tau proliferation and cognitive decline.

In addition to replicating our previous findings, we investigated the early relationship of plasma pTau217 with Aβ PET through the more flexible lens of EXT. Previous studies have shown that pTau217 rises before global Aβ positivity occurs [[13], [14], [15]]. However, we hypothesize that EXT may allow for better temporal correspondence between the PET and plasma measures of Aβ prior to reaching traditional global Aβ positivity given our results that early Aβ deposits can be better detected using EXT [7].

## 2 Methods

### 2.1 Sample

We included 1118 cognitively unimpaired older adults (Clinical Dementia Rating=0, Mini-Mental State Exam 25–30, Logical Memory 6–18) from the Anti-Amyloid Treatment in Asymptomatic Alzheimer’s disease (A4) placebo arm (*n* = 580) and Longitudinal Evaluation of Amyloid and Neurodegeneration Risk (LEARN, *n* = 538) studies. The A4 Study was a Phase 3 clinical trial designed to evaluate whether solanezumab, a monoclonal antibody targeting monomeric Aβ, could slow cognitive decline in older adults at the stage of preclinical Alzheimer’s disease (AD) [16,17]. Participants were cognitively and functionally unimpaired at baseline but showed evidence of elevated amyloid (Aβ+) on screening PET imaging. Individuals classified as Aβ- at screening were invited to join the LEARN Study, an observational companion study tracking cognitive and functional outcomes using the same assessments as the A4 Study [16]. PET scans were reprocessed at Massachusetts General Hospital and 18 participants (n_LEARN_=15, n_A4_=3) were excluded due to poor image quality/registration, resulting in the final sample of 1118 participants.

Of these participants, 707 had follow-up FBP-PET scans available. Follow-up scans were removed for 35 participants due to a change in scanner mid-study at two sites, resulting in a total of 672 participants with follow-up FBP-PET scans (median length=4.96±0.90 years). All participants also underwent cognitive testing every 6 months, with a median follow-up duration of 5.5 ± 2.3 years. A subset of 246 participants (n_LEARN_=55, n_A4_=191) had at least a baseline tau-PET scan using flortaucipir (FTP), and 206 (n_LEARN_=49, n_A4_=157) had 2–4 scans over a median follow-up of 4.60±1.18 years.

The study was conducted in accordance with ethical standards, with approval obtained from institutional review boards at all participating sites. Written informed consent was provided by all participants and their study partners prior to data collection at each site, including explicit consent for data sharing. All data were deidentified.

#### 2.1.1 Diversity, equity and inclusion

The A4 and LEARN studies recruited participants from diverse regions, including clinical sites across the United States, Canada, Japan, and Australia. Women are well-represented in the present sample (61.1 % female-identifying). Efforts were made to increase recruitment of minoritized populations for A4 and LEARN, defined in this study as individuals with an ethnoracial background other than non-Hispanic white. In the combined A4 and LEARN sample, 10.0 % were from minoritized populations: 2.7 % Black or African American, 2.7 % Asian, 0.8 % American Indian or Alaskan Native, 1.0 % reporting more than one race, and 2.9 % identifying as Hispanic white. Analyses included biological sex as a covariate but did not adjust for race/ethnicity due to the relatively small sample sizes once subdivided by race/ethnicity.

### 2.2 PET acquisition and preprocessing

At each A4 site, Aβ-PET was acquired using [^18^F]-Florbetapir (FBP) and tau-PET with [^18^F]-Flortaucipir (FTP) [16]. Both tracers were administered (on separate days) via intravenous injection of the tracer at a standard dose of approximately 370 MBq (10 mCi), followed by a 50–70 min scan for FBP and a 75–105 min scan for FTP. The PET scans utilized a dynamic acquisition protocol, capturing multiple frames (4 × 5 min for FBP, 6 × 5 min for FTP) to assess tracer uptake. Image reconstruction employed iterative algorithms, with corrections applied for scatter, randoms, and attenuation to ensure high-quality and accurate imaging results [16]. Additional scanner information is provided in Supplemental Table S1. A4/LEARN images were reprocessed using the HABS PET pipeline [18,19]. Both FBP and FTP PET images were coregistered to each participant’s baseline MRI, which was processed and parcellated with Freesurfer v.7 [20,21].

#### 2.2.1 Aβ-PET processing

The average FBP standardized uptake value ratio (SUVR) was computed across a standard neocortical (NEO) aggregate of 42 regions from the Desikan-Killiany atlas [22], as previously reported and well-validated [12,[23], [24], [25], [26], [27], [28]]. Gaussian Mixture Modeling (GMM) was used to derive positivity thresholds for the NEO aggregate (SUVR+ threshold=0.743) and each of the 42 ROIs in the NEO aggregate (Supplementary Table S2/Fig. S1). Due to FBP’s increased non-specific binding in white matter, associated reduction in gray-to-white matter contrast relative to PiB, and issues with longitudinal reliability of the traditional whole cerebellum FBP reference region [[8], [9], [10]], we utilized a composite reference region that included both whole cerebellum and eroded cortical white matter [29,30]. All primary analyses were repeated with the common whole cerebellum reference region alone (Supplemental Figs. S2-S7, Table S3) and showed the same pattern of results but with weaker effect sizes.

#### 2.2.2 Aβ-PET spatial extent computation

Spatial extent (EXT) was computed as the percentage of the NEO aggregate that was FBP+ using the same approach described previously in HABS [7]. For an individual, each of the 42 ROIs in the NEO aggregate was classified as positive or negative relative to the ROI’s region-specific GMM threshold. EXT was then calculated as the sum of the number of voxels in FBP+ ROIs divided by the total number of voxels in the NEO aggregate. This was expressed as a percentage. EXT values ranged from 0 % (no FBP+ NEO ROIs) to 100 % (all FBP+ NEO ROIs).

A priori thresholds from HABS [7] for Aβ EXT positivity (7.3 % EXT) and widespread EXT (95.6 % EXT) were used to stage participants. These thresholds were derived previously [7] to allow room for false positive ROIs when classifying Aβ EXT positivity (≥7.3 % rather than >0 %) and false negative ROIs when classifying widespread EXT (>95.6 % rather requiring 100 %). This resulted in 3 EXT stages: EXT0 0 (0–7.2 %): no Aβ or insufficient Aβ for reliable PET detection; EXT1 (7.3–95.5 %): spreading Aβ; EXT2 (95.6–100 %): widespread neocortical Aβ.

To test EXT’s robustness and cross-sectional reliability against variability in each of the ROI thresholds, bootstrapped samples (*R* = 1000, with replacement) were generated to estimate 95 % confidence intervals around each ROI threshold. Resampled EXT values were generated (*n* = 1000) for each individual by random selection of new thresholds from the bootstrapped ROI threshold samples. We also used these resampled EXT values to derive secondary within-sample cutoffs for EXT positivity (EXT0 vs EXT1 cutoff) and widespread EXT (EXT1 vs EXT2), set at the upper limit of the 95 % confidence interval for individuals with an original EXT value of 0 % (7.4 %, compared to the *a priori* 7.3 %) and the lower limit for those with an original confidence interval of 100 % (89.3 %, compared to the *a priori* 95.6 %). While there was greater uncertainty near 100 % EXT in A4/LEARN than HABS, use of the within-sample EXT2 threshold resulted in more frequent EXT2 to EXT1 stage regressions, so we elected to keep the a priori stage thresholds.

#### 2.2.3 Tau-PET processing

FTP SUVR with a cerebellar grey matter reference region was calculated for two early tau aggregates: MTL (entorhinal, parahippocampal, amygdala) and temporal neocortex (nTEMP: inferior temporal, fusiform, middle temporal). Data were partial volume corrected (PVC) using the Geometric Transfer Matrix method [31]. Analyses were repeated using non-PVC FTP data, but the pattern of the results were the same.

### 2.3 pTau217 quantification

As previously described, baseline plasma samples were collected during screening from A4/LEARN participants using identical protocols [32] within three months of PET. Plasma pTau217 was quantified with an analytically validated ECL immunoassay developed by Eli Lilly and Company using a MesoScale (MSD) Sector S Imager 600 MM at the CAP-accredited, CLIA-certified Lilly Clinical Diagnostics Laboratory [32].

### 2.4 Cognition

The Preclinical Alzheimer Cognitive Composite (PACC) [33,34] was computed as the averaged z-scores of 4 tests (Mini Mental State Examination [35,36], Logical Memory Delayed Recall [37], Free and Cued Selective Reminding Test [38,39], Digit Symbol Substitution [[40], [41], [42], [43]]).

### 2.5 Statistical analysis

All analyses were conducted in *R,* version 3.6.0 [44]. T-tests and χ^2^-tests were used to assess demographic differences between EXT stages. Analyses first compared the distributions of EXT and SUVR as Aβ metrics both cross-sectionally and longitudinally. For analyses directly comparing EXT and SUVR, both variables were z-scored. Since EXT expresses spread of Aβ from 0 to 100 % of the neocortex, we used logistic growth modeling to estimate the relationship between EXT and SUVR. The asymptote was constrained to 100 %, and we selected the midpoint and logistic growth rate parameters with the lowest sum of squared error. Reliability of the EXT values was assessed by computing the average 95 % confidence intervals after resampling from bootstrapped ROI threshold samples. The frequency of changes in EXT stage after resampling was estimated as the total number of stage changes out of a possible 1000 resamples for each participant.

To assess EXT’s utility for early detection below traditional global Aβ PET thresholds, receiver operator characteristic (ROC) curve analyses were conducted using Aβ EXT or SUVR to predict whether SUVR- individuals (SUVR<0.743) would progress to SUVR+ within the next 5.5 years. We further evaluated correspondence between EXT and ptau217 in SUVR- individuals. First, we determined whether our results aligned with previous findings of rise in ptau217 below the SUVR threshold by using linear models to assess the baseline relationship between Aβ SUVR and baseline pTau217 in SUVR- individuals. Next, we examined the data longitudinally using linear mixed effects (LME) models to confirm that higher baseline pTau217 in SUVR- participants predicts future increase in Aβ SUVR. We then evaluated the EXT group*pTau217 interaction cross-sectionally and the EXT group*pTau217*Time longitudinally to evaluate whether the association between pTau217 and SUVR was present only for those with detectable Aβ (EXT1) and not in Aβ- individuals (EXT0). We then examined the relationship between pTau217 and EXT in the full sample to assess their correspondence across the full spectrum of Aβ burden. All models covaried for age, sex, education, and APOE ε4 status. LMEs included random intercepts.

Next, we used LME models to assess baseline EXT as a predictor of tau proliferation (MTL and nTEMP FTP SUVR) and cognitive decline (PACC) over time. All models included age, sex, years of education, and their interactions with time as covariates, and random participant intercepts and slopes. First, we evaluated the differences in tau and cognitive trajectories by baseline EXT stage. Next, we compared continuous baseline EXT and SUVR as metrics to quantify the effect of Aβ on tau and cognition over time. For each outcome, we conducted 3 models: two independent models testing baseline EXT or baseline SUVR as predictors, and one combined model including both baseline EXT and SUVR. Comparison of the Aβ*time effect sizes (partial η^2^) in each model and Akaike’s Information Criterion (AIC) were computed to determine whether EXT or SUVR provided a stronger prediction of changing tau and cognition or if both metrics contributed. LME models were repeated within two subsamples (EXT0|1, EXT<50 %) to test the predictive utility of EXT and SUVR at different stages of amyloidosis.

## 3 Results

### 3.1 Sample characteristics

Sample characteristics grouped by baseline EXT stage are shown in Table 1 for both the full sample and tau-PET subsample. The age, the frequency of APOE4 carrier status, and pTau217 concentration increased from EXT0 to EXT1 to EXT2. The Aβ distribution was notably different from HABS, with a lower proportion of individuals categorized as EXT0 (P_A4LEARN_=40.4 %, P_HABS_=63.8 %), and a higher proportion in EXT1 (P_A4LEARN_=37.6 %, P_HABS_=17.8 %) and a slightly higher proportion of EXT2 (P_A4LEARN_=22.0 %, P_HABS_=18.4 %).

**Table 1:** **Sample demographics.** Summary demographic data are shown for the full A4/LEARN sample and the subsample with tau PET. Data are mean (standard deviation) for continuous variable and count (percentage) for categorical variables. Group differences were evaluated using t tests for continuous variables and χ2 for categorical variables.

	**A4/LEARN (*n*=1118)**						**TAU subsample (*n*=246)**					
	**EXT0**	**EXT1**	**EXT2**	**EXT0 vs 1**	**EXT1 vs 2**	**EXT0 vs 2**	**EXT0**	**EXT1**	**EXT2**	**EXT0 vs 1**	**EXT1 vs 2**	**EXT0 vs 2**
**n**	452	420	246				63	113	70			
**Study, n ( %) LEARN**	405 (89.6 %)	130 (31 %)	3 (1.2 %)	<0.001	<0.001	<0.001	47 (74.6 %)	8 (7.1 %)	0 (0 %)	<0.001	0.057	<0.001
**Age, years**	70.3 (4.27)	71.1 (4.43)	73.2 (5.38)	0.016	<0.001	<0.001	69.4 (3.67)	71.3 (4.88)	72.5 (4.79)	0.007	0.085	<0.001
**Education, years**	16.8 (2.57)	16.6 (2.71)	16.5 (3.13)	0.244	0.937	0.281	16.8 (2.84)	16 (2.64)	15.8 (2.81)	0.060	0.668	0.038
**Sex, n ( %) female**	275 (60.8 %)	268 (63.8 %)	140 (56.9 %)	0.404	0.093	0.353	36 (57.1 %)	75 (66.4 %)	40 (57.1 %)	0.292	0.272	1.000
**APOE, n ( %) carrier**	94 (20.8 %)	213 (50.7 %)	159 (64.6 %)	<0.001	<0.001	<0.001	13 (20.6 %)	61 (54 %)	50 (71.4 %)	<0.001	0.028	<0.001
**Bl NEO FBP SUVR**	0.673 (0.028)	0.786 (0.059)	0.964 (0.076)	<0.001	<0.001	<0.001	0.674 (0.030)	0.794 (0.057)	0.957 (0.080)	<0.001	<0.001	<0.001
**Bl SUVR status, n ( %) SUVR+**	0 (0 %)	298 (71 %)	246 (100 %)	<0.001	<0.001	<0.001	0 (0 %)	86 (76.2 %)	70 (100 %)	<0.001	<0.001	<0.001
**pTau217 available, n ( %)**	437 (96.7 %)	407 (96.9 %)	235 (95.5 %)				62 (98.4 %)	112 (99.1 %)	66 (94.3 %)			
**pTau217 concentration**	0.148 (0.038)	0.204 (0.089)	0.348 (0.16)	<0.001	<0.001	<0.001	0.152 (0.031)	0.214 (0.11)	0.358 (0.16)	<0.001	<0.001	<0.001
**Cognitive follow-up length, years**	4.48 (1.58)	4.16 (1.65)	4.29 (1.48)	0.003	0.306	0.135						
**Tau PET follow-up length, years**							3.32 (1.69)	3.11 (1.94)	3.66 (1.79)	0.471	0.050	0.286
**Ethnoracial status, n ( %) minority**	44 (9.8 %)	45 (10.7 %)	13 (5.3 %)	0.981	0.206	0.359	3 (4.8 %)	16 (14.3 %)	2 (2.9 %)	0.199	0.159	0.48

### 3.2 EXT as a cross-sectional Aβ metric

**Fig. 1:**
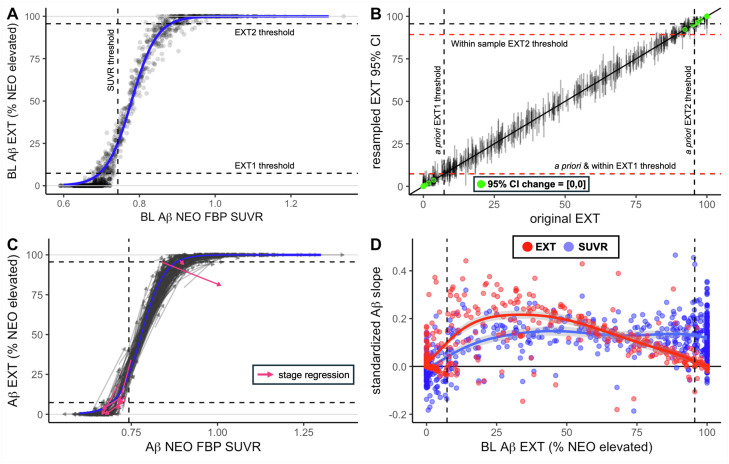
**Neocortical Aβ spatial extent (EXT) and average FBP SUVR as metrics for Aβ quantification.** A) Baseline SUVR (x-axis) versus EXT (y-axis) in A4/LEARN. The dashed vertical line indicates SUVR threshold (0.743 SUVR), and dashed horizontal lines indicate the *a priori* EXT1 detection threshold (7.3 % EXT, Aβ spreading phase), and EXT2 threshold (95.6 % EXT, widespread Aβ). The relationship between EXT and SUVR (blue curve) was fit with logistic growth modeling. Similar to HABS, individuals reached the EXT1 threshold prior to reaching typical SUVR positivity. Within the EXT1 stage, EXT and SUVR rose together as Aβ spread through the neocortex with EXT representing an expanded range relative to SUVR. As Aβ became widespread (EXT2), further increases in the amount of Aβ in the neocortex were quantified with SUVR. B) Cross-sectional reliability of the EXT metric was tested using bootstrapped GMM threshold resampling to evaluate the influence of variability in ROI thresholds. For each original EXT value (x-axis), 95 % confidence intervals of the resampled EXT values are shown (y-axis). Bootstrapped threshold variation caused minimal change in EXT value, and negligible change in EXT stage classification (2.6 %). Some participants near EXT extremes exhibited no variability after resampling EXT computation 1000 times (green dots). C) The longitudinal relationship between EXT and SUVR followed the baseline curve. Most participants remained within their baseline stage at follow-up or increased in stage (gray). Very few individuals (8/672) regressed in EXT stage at follow-up (pink). D) Both EXT and SUVR were standardized relative to their baseline distribution (z-score) and used to generate standardized EXT slopes (red) and SUVR slopes (blue) that are plotted against baseline EXT. Standardized Aβ slopes demonstrate that EXT changes more rapidly initially than SUVR but peaks and slows towards no change as Aβ approaches widespread EXT while SUVR slope remains elevated.

Fig. 1A displays the baseline relationship between EXT and SUVR as measures of Aβ in A4/LEARN. This relationship was fitted to a logistic growth model (midpoint=0.779 ± 0.002, logistic growth rate=0.0295 ± 0.001). Consistent with previous findings in HABS, the use of EXT condensed the dynamic range of the upper and lower ends of the Aβ distribution (EXT0 and EXT2) and expanded the dynamic range in the potentially critical spreading stage (EXT1).

#### 3.2.1 EXT0 (0–7.3 % EXT)

All EXT0/EXT- participants were also SUVR-, but many SUVR- participants (*n* = 122, 21.3 %) were EXT1. SUVR in EXT0 participants (range=[0.59,0.73], *M* = 0.67, SD=0.03) was condensed to the short 0–7.3 % EXT range, translating to a 97.1 % reduction in variance (σ^2^_SUVRz_=0.052, σ^2^_EXTz_=0.001). A majority of EXT0 participants (65.7 %) shared the same 0 % EXT value (no elevated ROIs). Higher SUVR at baseline within EXT0 was significantly associated with subsequent decline in SUVR (*r*=−0.21, *p*=.002), reaffirming that subthreshold variance expressed by SUVR predominantly reflects noise rather than subthreshold Aβ.

#### 3.2.2 EXT1 (7.3–95.5 % EXT)

On average, participants reached EXT1 below the 0.743 SUVR threshold at 0.705 SUVR. Within EXT1, we observed a concerted rise in EXT and SUVR (*r* = 0.955, *p*<.001). There was less variance in SUVR (range=[0.67,0.95], *M* = 0.786, SD=0.059) than EXT within EXT1, translating to a 122 % expansion of variance (σ^2^_SUVRz_=0.228, σ^2^_EXTz_=0.505) related to the spread of Aβ throughout the neocortex.

#### 3.2.3 EXT2 (95.6–100 % EXT)

Participants plateaued as they approached 100 % EXT at an average of 0.870 SUVR. Individuals in EXT2 had an extensive range of SUVR (range=[0.82,1.34], *M* = 0.964, SD=0.076) related to continued increase in Aβ burden after Aβ was detectable across the neocortex. This translates to a 99.7 % reduction in the variance expressed with SUVR compared to EXT (σ^2^_SUVRz_=0.388, σ^2^_EXTz_=0.001).

#### 3.2.4 Cross-sectional EXT reliability

We evaluated the cross-sectional reliability of EXT in response to variation in ROI thresholds after bootstrapped GMM recomputation by computing a range and 95 % CI of resampled EXT values for each subject and taking the average of the 2.5 % and 97.5 % bounds. Changes in EXT using the bootstrapped ROI thresholds were minimal (range: [−1.92,2.31], see Fig. 1B) and resulted in changes in EXT stage only 2.6 % of the time. However, it is also notable that the A4/LEARN sample size (*n* = 1118) was larger than HABS (*n* = 261), reducing variation in the bootstrapped ROI thresholds. Constraining the sample size to 261 during resampling for the bootstrapped GMM analyses resulted in a greater difference between resampled EXT and original EXT values (95 % CI: [−4.32,4.06]) and more frequent (8.4 %) change in EXT stage. These findings are consistent with the expectation that reliability would be poorer in A4/LEARN than HABS due to multiple factors including increased non-specific binding with FBP relative to PiB, site and scanner effects, and other sources of variability inherent in clinical trial data. However, the results also suggest that using a large sample to generate the ROI thresholds needed to compute EXT helps overcome these limitations.

### 3.3 EXT as a longitudinal Aβ measure

A total of 672 participants had longitudinal FBP follow-up over a median of 4.96±0.90 years (see Fig. 1C). As shown in Fig. 1C, participants most frequently remained in the same EXT stage (*n* = 509, *P* = 75.7 %), but increases in stage (*n* = 155, *P* = 23.1 %) were far more frequent than regression to a lower stage (*n* = 8, *P* = 1.2 %, χ^2^ = 132.6, *p*<.001). EXT was less prone to negative slopes than SUVR (9.2 % vs 23.7 %). Even after standardization (Fig. 1D), negative EXT slopes were of lesser magnitude (M_EXT_= −0.0240, SD_EXT_=0.0335) than negative SUVR slopes (M_SUVR_= −0.0429, SD_SUVR_=0.0351, *t* = 3.573, *p*<.001). As shown in Fig. 1D, the standardized rate of Aβ change was initially faster with EXT (EXT_0–50 %_: *M* = 0.076, SD=0.116, COV=1.515) than SUVR (*M* = 0.034, SD=0.090, COV=2.665) but because the rate of EXT change slowed as EXT approached 100 % (EXT_50–100 %_: *M* = 0.042, SD=0.068, COV=1.619) it was overtaken by SUVR (*M* = 0.127, SD=0.080, COV=0.630). Consistent with expectation for a multi-site study using FBP, EXT decreased over time in a greater proportion of observations in A4/LEARN (*P* = 9.2 %) than previously reported for HABS (*P* = 6.0 %, *p*=.049), but resulted in a similarly low frequency of stage regressions (P_A4LEARN_=1.2 %, P_HABS_=0.9 %).

### 3.4 Early Aβ detection with EXT

Classifying Aβ positivity using EXT rather than SUVR would change positivity from Aβ- to Aβ+ in 11.0 % of the overall A4/LEARN sample and 21.4 % of the SUVR- participants. Longitudinal FBP was available for 57 of these EXT+/SUVR- participants and 50 continued increasing in Aβ over time when measured using SUVR and EXT slope.

**Fig. 2:**
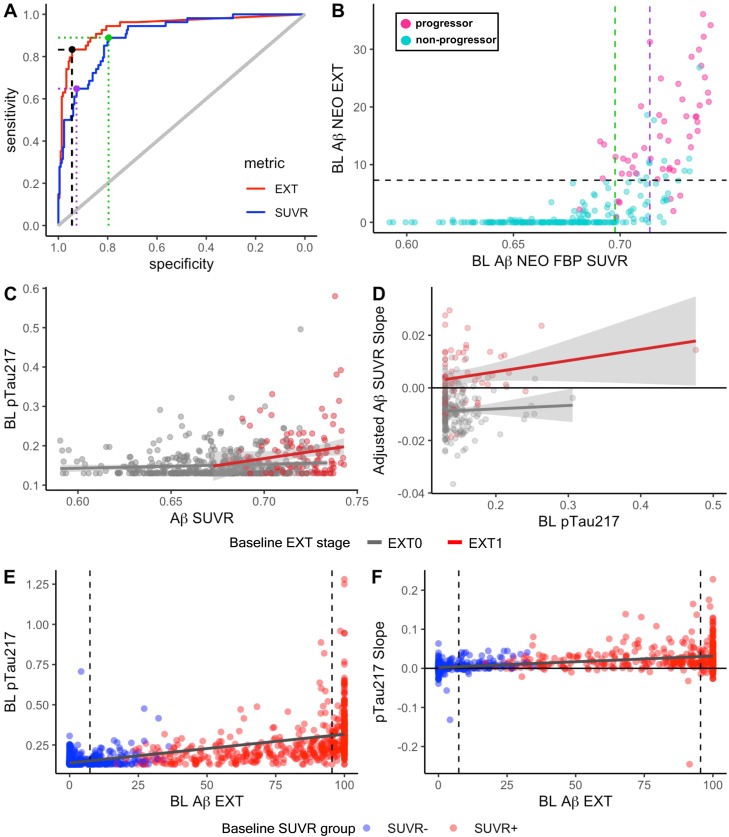
**Early detection of amyloidosis in the SUVR- subsample and associations with plasma ptau217.** A) Receiver operator characteristic (ROC) analyses were conducted using baseline Aβ EXT (red) or SUVR (blue) to predict progression of SUVR- individuals to SUVR positivity over the 5.5-year follow-up. EXT outperformed SUVR (AUC_EXT_=0.94 [CI: 0.90–0.98], AUC_SUVR_=0.89 [CI: 0.86–0.94], DeLong’s *Z* = 2.83, *p*=.005) as a continuous variable. The optimal EXT threshold (7.3 %, black dot) was the same in A4/LEARN as the previously reported threshold in HABS. The optimal thresholds for SUVR were the overall maximum Youden index (0.698 SUVR, SE=89 %, SP=80 %, PPV=52 %, green dot) and the maximum Youden Index with specificity constrained to >90 % (0.714 SUVR, purple). B) To visualize the sensitivity and specificity of the ROC thresholds, baseline FBP SUVRs are plotted against baseline EXT in initially SUVR- participants and colored by progressor status. The horizontal dashed line represents the optimal EXT threshold (7.3 %) such that progressors (pink) above the line are true positives detected by extent (*n* = 45) and those below are false negatives (*n* = 9) missed by extent resulting in 83 % sensitivity. The EXT+ threshold misses non-progressors in turquoise. The vertical dashed lines represent potential lowered SUVR thresholds, at the overall maximum Youden index (green) and the SP>90 % maximum Youden Index (purple). While lowering the SUVR threshold would capture some of the progressors (pink) detected with EXT, it would also increase the number of false positives that do not progress to SUVR+ after 5 years (turquoise). C) A scatterplot depicts the cross-sectional relationship at baseline between global Aβ SUVR and pTau217 concentration, grouped by Aβ EXT stage (EXT0=gray, EXT1=red). While previous studies have indicated a subthreshold relationship between global Aβ SUVR and pTau217 below PET detection thresholds, we see that this subthreshold association is driven by an plasma-PET association in individuals that are PET+ when using EXT. D) Extracted Aβ SUVR slopes, adjusted for age, sex, years of education, and APOE ε4 carrier status, are plotted against baseline pTau217 and grouped by baseline Aβ EXT stage (EXT0=gray, EXT1=red). In alignment with the linear mixed effects models reported in the text, we observe that higher pTau217 is associated with faster Aβ accumulation (higher SUVR slope) but only when participants were also PET+ based on Aβ EXT (EXT1, red) at baseline. E) Looking across the full Aβ continuum (including both SUVR- in blue and SUVR+ in red), a scatterplot captures the cross-sectional association between baseline Aβ EXT and plasma pTau217 concentration. Vertical dashed lines indicate predefined EXT stage thresholds. Baseline pTau217 concentrations increased with greater Aβ EXT, with the clearest elevations in pTau217 observed as participants approach widespread neocortical Aβ in the EXT2 stage. F) Across the full Aβ continuum (SUVR-: blue, SUVR+:red), a longitudinal association is shown between baseline Aβ EXT and extracted plasma pTau217 slopes. Due to high test-retest variability for plasma ptau217, pTau217 slopes are shown only for participants with 3 or more ptau217 assessments to improve visualization. Higher baseline Aβ EXT was associated with larger subsequent increases in pTau217, again with changes predominant as participants approach widespread Aβ EXT.

We next evaluated EXT’s ability to predict progression from SUVR- to the gold standard SUVR+ at follow-up. As shown in Fig. 2A, continuous baseline EXT was a better predictor of progression from SUVR- to SUVR+ within the next 5 years (AUC=0.94 [CI: 0.90–0.98]) than continuous baseline SUVR burden (AUC=0.89 [CI: 0.86–0.94], DeLong’s *Z* = 2.83, *p=*.005). The *a priori* HABS 7.3 % EXT threshold also provided optimal prediction of progression to SUVR+ in A4/LEARN (SE=0.83, SP=0.94, PPV=0.79). Selecting a lowered SUVR threshold by maximizing the Youden Index (0.698 SUVR, Fig. 2A green dot, Fig. 2B green dashed line) also enabled high sensitivity (SE=89 %, SP=80 %) but increased the number of false positives to the degree that the positive predictive value was barely above chance (PPV=52 %). The SUVR threshold that came closest to EXT’s performance (0.714 SUVR, Fig. 2A purple point, Fig. 2B purple dashed line) still fell short of EXT’s prediction of progression to SUVR+ (SE=65 %, SP=93 %, PPV=69 %). Furthermore, if we considered initially SUVR- whose SUVR increased at follow-up without reaching the gold standard SUVR threshold, the positive predictive value increased to 88 % for EXT but remained low for SUVR (0.698 threshold: PPV=62 %, 0.714 threshold: PPV=71 %).

### 3.5 pTau217 and earlier detection of Aβ

Since pTau217 has previously been implicated as an early indicator of emerging Aβ pathology even below global PET thresholds [[13], [14], [15]], we initially focused our analyses on the SUVR- subgroup to more specifically interrogate the earliest detectable relationship between pTau217 and Aβ PET. At baseline, we observed a positive association between higher pTau217 concentration and greater SUVR (*β*=0.160, *SE*=0.040, *p*<.0001). However, when we introduce EXT group into the model, the baseline association of pTau217 with SUVR differs significantly between the EXT0 and EXT1 groups (pTau217*EXT group: *β*=0.162, *SE*=0.075, *p*=.0324, Fig. 2C). When we break down the interaction by looking within EXT group, higher baseline SUVR corresponds with higher baseline pTau217 only in EXT1 (*β*=0.197, *SE*=0.046, *p*<.0001); there is no relationship within EXT0 (*β*=0.0447, *SE*=0.055, *p*=.421). These results indicate that the EXT1 group drives the significant relationship seen with SUVR in the SUVR- subsample. When directly comparing continuous EXT and ptau217, we observed a similar pattern of association between EXT and ptau217 in the EXT1 but not the EXT0 stage (Supplemental Fig. S8A)

These findings held when examined longitudinally with a linear mixed effects model (pTau217*Time*EXT group). The three-way interaction showed that greater baseline pTau217 predicted higher rates of SUVR increase over time in EXT1 relative to EXT0 (*β*=0.0553, *SE*=0.012, *p*<.0001). Breaking down the interaction by EXT groups, we saw positive SUVR change in EXT1 but no change in EXT0 (EXT1: *β*=0.0674, *SE*=0.012, *p*<.0001; EXT0: *β*=0.0122, *SE*=0.0086, *p*=.158; Fig. 2D), supporting that elevated pTau217 concentration in individuals below traditional Aβ PET positivity thresholds may be capturing the earliest deposits Aβ also detected by Aβ EXT. Similarly, when using baseline ptau217 to predict change in EXT, greater baseline ptau217 concentration was associated with faster change in EXT only in individuals who were in the EXT1 stage at baseline (Supplemental Fig. S8B).

Lastly, we also examined each baseline Aβ metric as predictors of change in ptau217 concentration over time in the SUVR- subsample. Both EXT (*β*=0.023,*SE*=0.0004*, p*<.001) and SUVR (*β*=0.010*, SE*=0.0003, *p*<.001) were significant predictors of ptau217 change but with a greater effect size for EXT (η^2^ = 0.18) than SUVR (η^2^ = 0.08).

### 3.6 Relationship between EXT, SUVR, and pTau217 across the full sample

We next examined the association between pTau217 and Aβ PET measures across the full sample. At baseline, pTau217 concentration was positively associated with both EXT (β=21.821, *SE*=0.989, *p*<.0001, Fig. 2E) and global SUVR (*β*=0.0713, *SE*=0.0027, *p*<.0001). Longitudinally, both higher baseline EXT (EXT*Time: *β*=0.010, *SE*=0.0001, *p*<.0001, η^2^ = 0.11; Fig. 2F) and higher baseline SUVR (SUVR*Time: *β*=0.010, *SE*=0.0002, *p*<.0001, η^2^ = 0.11) provided similar association with increasing plasma pTau217 over time. Notably, the strength of both the cross-sectional and longitudinal associations between pTau217 and Aβ was greatest in those with widespread Aβ (EXT stage 2) and those with high EXT approaching Stage 2. Together, these results indicate that while pTau217 may detect early focal Aβ deposition below conventional SUVR thresholds that corresponds with elevated EXT, its association with Aβ is most robust once pathology is extensive.

### 3.7 Tau proliferation and cognitive decline across Aβ EXT stages

**Fig. 3:**
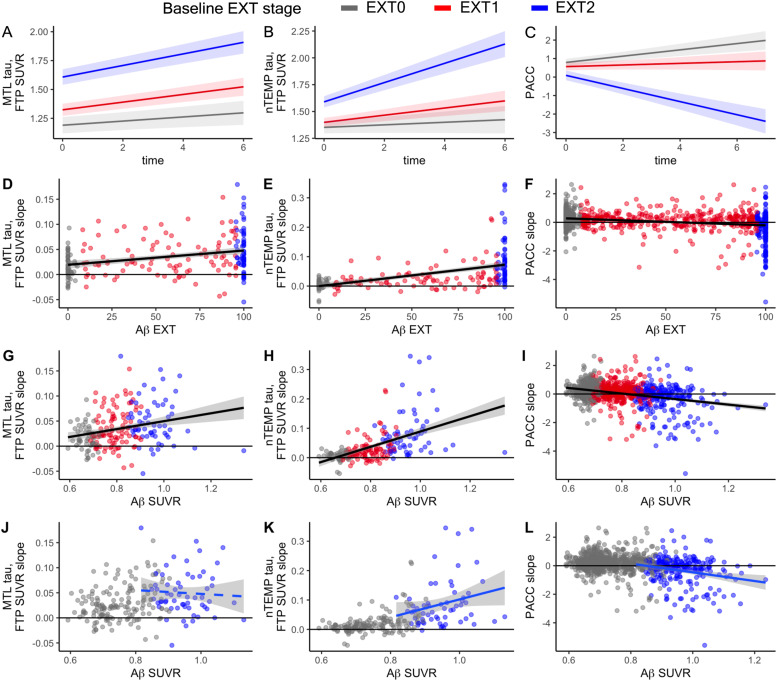
**Relationships of baseline EXT and SUVR with tau and cognition change.** In the top row, estimated marginal means from the linear mixed effect models of the baseline EXT stage*time effect on A) MTL tau, B) nTEMP tau and C) PACC are shown for the average participant (71.8 year-old female with 16.0 years of education). A) Slow but significant change of over time in MTL tau was observed in Aβ- individuals in EXT0. However, spreading Aβ in EXT1 was associated with faster MTL tau proliferation over time than EXT 0. Individuals with widespread Aβ individuals in EXT2 exhibited the fastest change in MTL tau over time. B) In contrast with MTL tau, EXT0 participants did not exhibit changing tau in the nTEMP. Increasing nTEMP tau over time was observed on average in EXT1 participants, but at a slow rate relative the more rapid proliferation seen in EXT2 participants. C) EXT2 is also associated with the fastest rate of decline on PACC. EXT1 was not associated with explicit decline but rather a lack of learning from prior exposures to the PACC thus diverging from EXT0 where practice effects contribute to an increase in PACC score over time. d-I) Scatterplots of extracted slopes for MTL tau (first column), nTEMP tau (2nd column), and PACC (3rd column) are plotted against baseline Aβ EXT (2nd row) or baseline Aβ SUVR (3rd row). Points are colored by Aβ EXT stage. Both Aβ EXT and SUVR are associated with future tau proliferation and cognitive declice, but their variance emphasizes detection of these associations at different Aβ stages (EXT1 for EXT, EXT2 for SUVR). J-L) The baseline Aβ SUVR scatterplots (G-I) are recoded to focus on the EXT2 group (blue) with widespread Aβ, plotting the linear regression line only within this stage. This helps to explain why SUVR was the superior predictor for nTEMP tau and PACC while EXT was superior for MTL tau. A single outlier with the highest Aβ SUVR (SUVR>1.3, see G-I) and no evidence of tau proliferation was removed to avoid underestimation of the association between continued increases in the concentration of Aβ with SUVR and future tau proliferation and PACC decline.

Higher baseline Aβ EXT stage corresponded with increasing MTL and nTEMP tau proliferation, and worsening PACC performance (Fig. 3A-C). Even in Aβ- individuals in EXT0, MTL tau increased significantly over time (*β*=0.018, *SE*=0.006, *p*=.003), whereas nTEMP tau did not (*β*=0.012, *SE*=0.008, *p*=.131) and PACC exhibited a practice effect (*β*=0.162, *SE*=0.034, *p*<.001). Individuals in EXT1 exhibited accelerated tau proliferation in the MTL relative to EXT0 (*β*=0.015, *SE*=0.007, *p*=.018) as well as the start of increasing tau in the nTEMP (*β*=0.022, *SE*=0.009, *p*=.015) and a lack of a practice effect for PACC (*β*= −0.126, *SE*=0.043, *p*=.003). Relative to EXT1 (estimated annualized slopes: MTL=0.033; nTEMP=0.034; PACC= −0.036), EXT2 individuals with widespread Aβ worsened faster over time on all 3 outcomes, with an observed annual change of 0.050 in MTL tau, 0.090 in nTEMP tau, and −0.363 in PACC score with statistically significant group difference on all measures (MTL: *β*=0.025, *SE*=0.007, *p*<.001; nTEMP: *β*=0.078, *SE*=0.010, *p*<.001; PACC: *β*= −0.525, *SE*=0.051, *p*<.001). Approximation of these EXT stages using SUVR (SUVR-/+/++ stages) yielded similar but slightly weaker effects (Supplemental Section S2, Fig. S9).

### 3.8 EXT vs. SUVR as predictors of tau proliferation and cognitive decline

We next assessed continuous baseline Aβ EXT and SUVR burden as predictors of future change in tau SUVR (MTL and nTEMP) and cognition (PACC) over time (Table 2, Fig. 3D-I). For all 3 outcomes, the best fitting model included both EXT and SUVR as contributing measures to baseline Aβ’s association with changing tau and cognition. However, in each case one of the two Aβ metrics dominated, with EXT more strongly associated with changing MTL tau and SUVR more strongly associated with changing nTEMP tau and PACC. Differences in which Aβ metric was superior were driven by each outcome measure’s association with SUVR once Aβ was widespread in the EXT2 stage. Within the EXT2 subsample, increasing SUVR was not associated with faster MTL tau proliferation (*β*= −0.007, *SE*=0.009, *p*=.396, Fig. 3J-L) but was associated with faster PACC decline (*β*= −0.345, *SE*=0.097, *p*<.001, Fig. 3L). Faster proliferation of nTEMP tau SUVR was also associated with higher baseline Aβ SUVR in the EXT2 subsample, though only after removing an outlier with high Aβ SUVR and limited nTEMP tau change (*β*=0.040, *SE*=0.018, *p*=.035, Fig. 3L).

**Table 2:** **Linear mixed-effects model results examining the predictive power of baseline amyloid burden (SUVR) and spatial extent (EXT) on cognitive decline (PACC) and tau accumulation (MTL and nTEMP)**. For each outcome, 3 models were performed. First, independent models were run to test the association between each Aβ metric at baseline on each outcome over time (Model 1: time*SUVR, Model 2: time*EXT). EXT and SUVR were standardized to allow for direct comparison of the β estimates and standard error between models. Effect sizes (partial η²) are reported to provide estimates of EXT and SUVR’s effect on each outcome and Akaike Information Criterion (AIC) provides a measure of overall model fit. Next, we conducted a combined model including the time*EXT and time*SUVR terms in the same model to see which measure was a better fit to the data. AIC for the combined model is also reported, allowing comparison against the independent models for EXT and SUVR to determine which model is the best fit (lowest AIC). This set of analyses were repeated within different subsamples representing different stages of amyloidosis, decreasing Aβ load from the full sample (All) to EXT0|1 to EXT<50 %. The model with the lowest AIC is reported in bold for each outcome/sample.

**Outcome**		**Independent Models**						**Combined Models**			
	**Sample**	**Metric**	**β**	**SE**	**p**	**η^2^**	**AIC**	**β**	**SE**	**p**	**AIC^a^**
**PACC**	**All**	SUVR	−0.218	0.019	<0.001	0.12	44,465	−0.301	0.048	<0.001	**44,459**
		EXT	−0.187	0.019	<0.001	0.09	44,511	0.090	0.049	0.065	
	**EXT0|1**	SUVR	−0.124	0.029	<0.001	0.03	**33,471**	−0.028	0.089	0.751	33,473
		EXT	−0.097	0.022	<0.001	0.03	33,471	−0.077	0.067	0.251	
	**EXT<50 %**	SUVR	−0.072	0.052	0.168	0.004	24,764	0.105	0.087	0.227	24,761
		EXT	−0.146	0.055	0.008	0.01	**24,761**	−0.236	0.092	0.011	
**MTL TAU**	**All**	SUVR	0.011	0.003	<0.001	0.07	−770	−0.003	0.006	0.602	**−773**
		EXT	0.013	0.003	<0.001	0.10	−758	0.016	0.006	0.010	
	**EXT0|1**	SUVR	0.012	0.005	0.013	0.04	−787	−0.008	0.015	0.600	−787
		EXT	0.010	0.003	0.005	0.06	**−791**	0.015	0.011	0.171	
	**EXT<50 %**	SUVR	0.010	0.008	0.177	0.02	−570	−0.018	0.013	0.188	−577
		EXT	0.018	0.007	0.010	0.09	**−579**	0.032	0.012	0.011	
**nTEMP TAU**	**All**	SUVR	0.031	0.004	<0.001	0.25	−1021	0.025	0.008	0.002	**−1027**
		EXT	0.030	0.004	<0.001	0.22	−1001	0.007	0.008	0.421	
	**EXT0|1**	SUVR	0.024	0.004	<0.001	0.17	−1062	0.004	0.014	0.758	−1060
		EXT	0.018	0.003	<0.001	0.19	**−1064**	0.015	0.010	0.147	
	**EXT<50 %**	SUVR	0.018	0.006	0.003	0.10	−803	−0.012	0.010	0.219	−813
		EXT	0.026	0.005	<0.001	0.24	**−815**	0.035	0.009	0.000	

#### 3.8.1 EXT vs. SUVR as predictors during early Aβ stages

Removing EXT2 participants to focus on earlier stages of amyloidosis (EXT0|1, Table 2) resulted in EXT alone providing the best fitting model for predicting change over time in both MTL (AIC_EXT MTL_= −791< AIC_SUVR MTL_= −787=AIC_EXT+SUVR MTL_= −787) and nTEMP tau (AIC_EXT nTEMP_= −1064< AIC_SUVR nTEMP_= −1062<AIC_EXT+SUVR MTL_= −1060). Models predicting PACC in EXT0|1 favored neither EXT nor SUVR, but subsetting even earlier (EXT<50 %, Table 2) resulted in a weak (*η*^2^=0.01) but significant association between EXT and PACC over time but not Aβ SUVR.

## 4 Discussion

Our findings support the utility of EXT as a robust and informative measure of Aβ pathology, particularly for detecting early neocortical Aβ spread in preclinical AD. Replication in the independent A4/LEARN cohort using FBP provides support for the generalizability of EXT, which showed high cross-sectional reliability and longitudinal stability. Compared to traditional global SUVR, EXT offered enhanced sensitivity to early Aβ deposition and improved prediction of MTL tau proliferation. While traditional global SUVR was superior for predicting tau proliferation in nTEMP and cognitive decline in the full A4/LEARN sample, EXT was more informative when focusing on those for whom Aβ was still spreading throughout Aβ-vulnerable neocortex. Overall, these findings support the specialized utility of EXT for studying the earliest changes in preclinical AD.

In addition to replicating our findings in an independent cohort, we sought to evaluate whether the EXT approach we previously developed in the HABS sample using PiB would be robust to the increased noise associated with a multi-site study employing FBP, a historically more accessible ^18^F-labeled radiotracer prone to higher non-specific binding in white matter [8,9]. Despite concerns that additional sources of noise would render the EXT metric unreliable, we observed excellent cross-sectional and longitudinal reliability and robust EXT-based Aβ staging. Two important factors contributed to this reliability and should be kept in mind for implementation in other samples: the large sample size and use of a composite reference region. The large sample size of A4/LEARN compensated for the reduced gray-to-white matter contrast and signal dynamic range differences between FBP and PiB by allowing for robust derivation of ROI thresholds using GMM. Smaller samples, particularly those with limited inclusion of individuals in early stages of amyloidosis, may encounter greater difficulty in deriving robust ROI thresholds and thus less reliable EXT measures. We also observed superior EXT reliability when using a composite reference region for FBP, rather than the conventional whole cerebellum reference region, that yielded less uncertainty around ROI positivity thresholds. The composite reference region was developed to improve longitudinal measurement of SUVR change, combining an eroded ROI of the more longitudinally stable cerebral white matter with FBP’s conventional but less longitudinally stable whole cerebellum reference region to reduce uncertainty about whether SUVR changes reflect changes in Aβ in the target region or changes in non-specific binding in the reference region [29,45]. We found that using the composite reference region improved cross-sectional EXT reliability in addition to improving longitudinal reliability, and strengthened the association between baseline EXT and future tau proliferation and cognitive decline. This suggests that the inclusion of eroded cerebral white matter in the reference region may help to mitigate the impact of partial volume effects from FBP’s high non-specific binding in cerebral white matter on target regions, though kinetic modeling is necessary to properly evaluate this possibility.

One of the greatest advantages conferred by EXT in our previous publication [7] was its marked improvement in early detection of Aβ, below typical global positivity thresholds. Individuals with SUVRs just below the global positivity threshold are typically a mix of those with early regional Aβ deposits and Aβ- individuals with high non-specific binding or other sources of noise, such that simply lowering global positivity thresholds results in high false positive rates, as shown in the present study. Many studies have leveraged longitudinal PET [30,[46], [47], [48]] or CSF Aβ measures [[49], [50], [51], [52], [53]] to differentiate early subthreshold Aβ from noise and have established that deleterious effects of Aβ on tau proliferation [30,47,52] and cognitive decline [30,46,[48], [49], [50], [51], [52]] begin below global positivity thresholds. In both our initial HABS study and the present findings from A4/LEARN, EXT distinguished early Aβ deposits from noise below the global positivity threshold without the need for additional longitudinal data. Although we observed a higher false positive rate in A4/LEARN than HABS, as expected for a multisite FBP study, EXT still sensitively predicted future global positivity with fewer false positives than a lowered SUVR threshold. Computing EXT across the neocortex allows flexibility in early Aβ detection and avoids potential selection bias to a particular anatomic phenotype introduced by other early detection approaches focused on a subset of commonly early-accumulating regions [51,[54], [55], [56], [57]]. Instead, EXT enables inclusive detection of focal positivity predictive of downstream pathology that may allow us to better target individuals for inclusion in clinical trials.

By leveraging EXT’s sensitivity to early amyloidosis, we were able to see correspondence between Aβ SUVR and pTau217 in traditionally Aβ- individuals, in contrast to previous findings reporting a rise in pTau217 concentration below traditional SUVR-based Aβ positivity thresholds [[13], [14], [15]]. The use of EXT appears to mitigate the temporal lag between early elevation in pTau217 and detection of Aβ deposition with PET, supporting the possibility of a biological link between the early spatial spread of Aβ and early elevation in pTau217. Additionally, analyses across the full cohort demonstrate that while modest increases in ptau217 concentration may begin below traditional global SUVR thresholds ptau217 becomes most clearly elevated as individuals approach widespread neocortical Aβ EXT in EXT Stage 2. This is consistent with previous evidence that pTau217 correlates best with PET at higher SUVR [15], and reinforces that the spatial information captured by EXT provides biologically relevant insight into the association between Aβ and other AD biomarkers.

Consistent with ample evidence of age-related tau proliferation in the MTL from autopsy [[58], [59], [60]] and in vivo PET studies [18,[61], [62], [63]], we observed slow but significant tau change in the MTL in Aβ- individuals within the EXT0 stage. Evidence of accelerated Aβ-related MTL tau proliferation began in the EXT1 spreading stage. In contrast, tau proliferation in the nTEMP was not observed in Aβ- EXT0 participants, and evidence of tau proliferation during the EXT1 spreading stage was minimal compared to the rapid proliferation observed after Aβ was widespread in EXT2. Our findings align with growing evidence that Aβ acts as a global permissive factor necessary for the spread of tau into the neocortex [64,65]. These findings further support the idea of targeting individuals in EXT1 for preclinical intervention trials aimed at preventing the downstream propagation of tau and cognitive impairment.

In both our previous work in HABS [7] and the current work in A4/LEARN, baseline EXT was a better predictor of future MTL tau proliferation than SUVR. However, results contrasted between cohorts for nTEMP tau and PACC, with SUVR outperforming EXT in A4/LEARN. Multiple individuals in HABS with high Aβ and low tau may represent rare cases of individuals resistant to tau pathology and potentially contributed to a dampened linear association between SUVR and nTEMP tau and PACC. Tau resisitant individuals may be best understood in future studies through independent analysis as a distinct entity from typical AD. Removal of one similar individual from A4/LEARN allowed for detection of a continued association between nTEMP tau change and further increases in concentration via SUVR once Aβ is widespread throughout the neocortex. This is consistent with evidence that Aβ also serves as a local facilitator [[64], [65], [66]] in addition to serving as a global permissive factor for tau proliferation. However, when we excluded all EXT2 individuals, EXT was a better predictor of nTEMP tau proliferation and cognitive decline than SUVR. This suggests complementary roles for EXT and SUVR dependent on disease stage, with EXT conferring advantages in the earliest stage and SUVR being of greater utility later.

### 4.1 Limitations

While converging results in our prior work and the present study support that the current neocortical spatial extent approach is sufficient for research aimed at understanding the importance of the spatial extent of Aβ pathology in the development of Alzheimer’s disease, additional research is needed to improve its feasibility for use as screening criteria in clinical trials. At present, the study-specific ROI thresholds necessary for spatial extent computation can only be derived after a large sample (*n* > 200) has already been collected due to the impact that differences in sample, scanner, tracer, and image processing pipelines have on SUVR. Scanner and site identifiers were not available at the subject level due to study blinding and deidentification procedures. Future research in other samples will seek to evaluate the impact of scanner differences on regional elevations and spatial extent accuracy.

To enable clinical trials to use spatial extent at screening to target individuals with early Aβ, study-specific ROI thresholds would need to be available *a priori*. Ongoing research aimed at improving understanding of spatial variations in non-specific binding and other sources of noise is underway to develop an *a priori* algorithm for measuring spatial extent for clinical trial applications. Differences in ethnoracial composition and biomarker profiles may also affect the optimal thresholds or ROI-specific signal characteristics, though current sample sizes are insufficient to rigorously evaluate these factors. Addressing these gaps will require more diverse and representative cohorts in future work. Future research will also evaluate whether more specific cognitive metrics of executive function and learning may enhance sensitivity to subtle cognitive changes associated with the early stages of spreading Aβ pathology.

### 4.2 Conclusions

Our findings show that Aβ EXT reliably detects the earliest cortical Aβ spread, before traditional global PET thresholds, and more accurately identifies individuals who will progress and accumulate MTL tau. By pinpointing individuals toward the beginning of Aβ spread, EXT offers a powerful tool to enrich prevention trials and accelerate evaluation of early-stage therapeutic efficacy in preclinical AD. Moving forward, we hope to apply EXT in clinical trial settings, particularly in the context of monoclonal anti-Aβ therapies, to evaluate whether spatial reduction of Aβ may help develop a better understanding of treatment efficacy. Given the increasing emphasis on intervention at the earliest stages of AD pathology, EXT may serve as a valuable biomarker both for cohort selection and monitoring therapeutic response.

## Sources of funding

This work was supported by a K01 Career Development Award from the National Institute on Aging (Farrell, 1K01AG083062). The A4 Study was funded by a public-private-philanthropic partnership, including funding from the National Institutes of Health-National Institute on Aging (R01 AG063689, U19AG010483 and U24AG057437), Eli Lilly and Company, Alzheimer's Association, Accelerating Medicines Partnership, GHR Foundation, an anonymous foundation, and additional private donors, with in-kind support from Avid Radiopharmaceuticals, Cogstate, Albert Einstein College of Medicine and the Foundation for Neurologic Diseases. The companion observational Longitudinal Evaluation of Amyloid Risk and Neurodegeneration (LEARN) Study was funded by the Alzheimer's Association (LEARN-15–338,729) and GHR Foundation.

## Consent statement

All participants provided informed consent. All experimental procedures were performed in ethical accordance with the Declaration of Helsinki and were approved and monitored by the local Institutional Review Boards.

## Declaration of generative AI and AI-assisted technologies in the writing process

At the analysis stage, ChatGPT was used as an aid to troubleshoot and refine R scripts, with thorough oversight from the authors of any AI-generated code. At the writing stage, ChatGPT was used to review the authors’ original drafts for conciseness and readability and any AI-suggested edits were reviewed by the authors prior to inclusion.

## Data sharing

Original data for A4 and LEARN are stored publicly on synapse.org. FBP and FTP scans were reprocessed in house to generate SUVR and spatial extent and are available upon request.

## Declaration of interest

E.G.T., G.D.C.M., J.A.B., J.C.P., R.F.B., H.I.L.J., M.J.P., and M.E.F. have no disclosures. B.J.H. has served as a paid consultant for Biogen, Eisai, and Roche. B.C.H. has received research support from Analysis Group, Celgene, Bristol-Myers Squibb, Verily Life Sciences, Merck-Serono, Novartis and Genzyme. R.A.S. has served as a paid consultant for Abbvie, AC Immune, Acumen, Alector, Biohaven, Bristol-Myers-Squibb, Janssen, Ionis, Prothena, and Roche. She has received research support as an investigator for Eli Lilly, and Eisai public private partnership clinical trials. K.A.J. has served as a paid consultant for Janssen, Merck and Novartis. He is a site coinvestigator for Eli Lilly/Avid, Pfizer, Janssen Immunotherapy. He has spoken at symposia sponsored by Janssen Alzheimer’s Immunotherapy and Pfizer. These relationships are not related to the content in the manuscript.

## CRediT authorship contribution statement

**Emma G. Thibault:** Writing – review & editing, Writing – original draft, Methodology, Investigation, Formal analysis. **Grace Del Carmen Montenegro:** Writing – original draft, Formal analysis. **J․Alex Becker:** Writing – review & editing, Investigation. **Julie C․ Price:** Writing – review & editing, Supervision, Investigation. **Brian C. Healy:** Writing – review & editing, Methodology, Investigation, Formal analysis. **Bernard J. Hanseeuw:** Writing – review & editing, Investigation. **Rachel F. Buckley:** Writing – review & editing, Investigation, Conceptualization. **Heidi I.L. Jacobs:** Writing – review & editing, Investigation, Conceptualization. **Michael J. Properzi:** Writing – review & editing, Investigation, Conceptualization. **Reisa A. Sperling:** Writing – review & editing, Supervision, Project administration, Methodology, Investigation, Funding acquisition, Conceptualization. **Keith A. Johnson:** Writing – review & editing, Supervision, Methodology, Investigation, Conceptualization. **Michelle E. Farrell:** Writing – review & editing, Writing – original draft, Supervision, Methodology, Investigation, Funding acquisition, Formal analysis, Data curation, Conceptualization.

## Declaration of competing interest

The authors declare the following financial interests/personal relationships which may be considered as potential competing interests:

Bernard J. Hanseeuw reports a relationship with Biogen, Eisai, and Roche that includes: consulting or advisory. Brian C. Healy reports a relationship with Analysis Group, Celgene, Bristol-Myers Squibb, Verily Life Sciences, Merck-Serono, Novartis and Genzyme that includes: funding grants. Reisa A. Sperling reports a relationship with Abbvie, AC Immune, Acumen, Alector, Biohaven, Bristol-Myers-Squibb, Janssen, Ionis, Prothena, and Roche that includes: consulting or advisory. Reisa A. Sperling reports a relationship with Eli Lilly, and Eisai that includes: funding grants. Keith A. Johnson reports a relationship with Janssen, Merck and Novartis that includes: consulting or advisory. Keith A. Johnson reports a relationship with Pfizer, Janssen Immunotherapy that includes: speaking and lecture fees. If there are other authors, they declare that they have no known competing financial interests or personal relationships that could have appeared to influence the work reported in this paper.

## Appendix Supplementary materials

Image, application 1
