# Supplementary material for: Amyloid spatial extent with florbetapir-PET for early detection of preclinical Alzheimer’s disease
Source: J Prev Alzheimers Dis. 2026 Mar 13;13(5):100529. doi: 10.1016/j.tjpad.2026.100529 (PMC12999308; doi:10.1016/j.tjpad.2026.100529)
Supplement: Supplementary file 1 [file mmc1.docx]

**Supplementary Materials**

**Section S1. Scanner information.** PET acquisition parameters were extracted from DICOM headers at each site. Scanner manufacturer, model, reconstruction algorithm, scatter correction, and randoms correction methods were harmonized across sites prior to analysis. Because sites varied across participating centers, scanner types are summarized below.

| **Siemens** | **n scans** | **Table S1. A4/LEARN study scanner types.** Scanner manufacturer and model are summarized across all PET acquisitions included in the present analyses. Minor iteration/subset variations within model families were not tabulated separately. PET images were processed using a standardized pipeline and harmonized quantification procedures across sites to minimize scanner-related variability. Counts reflect individual PET scans rather than unique participants. |
| --- | --- | --- |
| Biograph 20_mCT  Biograph 40_mCT  Biograph 64_mCT  Biograph128  Biograph128Edge_Vision 600  Biograph128_Vision 600 Edge  Biograph128_mCT  Biograph128_mCT 4R  Biograph16_Horizon 3R  Biograph16_TruePoint  Biograph20_mCT  Biograph20_mCT 3R  Biograph20_mCT 4R  Biograph40_TruePoint  Biograph40_mCT  Biograph40_mCT 4R  Biograph64_TruePoint  Biograph64_Vision 450  Biograph64_Vision 600  Biograph64_mCT  Biograph64_mCT 3R  Biograph64_mCT 4R  Biograph6_TruePoint  Biograph_mMR  SOMATOM Definition AS_mCT | 70  46  65  7  4  38  495  15  8  26  203  1  13  71  26  3  475  1  6  75  12  1  61  88  64 |  |
| **GE Medical Systems** |  |  |
| Discovery 600  Discovery 610  Discovery 690  Discovery 710  Discovery LS  Discovery MI  Discovery MI DR  Discovery RX  Discovery ST  Discovery STE | 98  62  159  236  74  38  52  50  228  391 |  |
| **Philips Medical Systems** |  |  |
| GEMINI TF Big Bore  GEMINI TF TOF 16  GEMINI TF TOF 64  Ingenuity CT  Ingenuity TF PET/CT  Vereos PET/CT | 32  43  96  89  153  10 |  |
| **Shimadzu Corp.** |  |  |
| EminenceSTARGATE | 21 |  |
| **Toshiba** |  |  |
| Aquiduo | 48 |  |
| **MiE** |  |  |
| SCIN | 30 |  |
| **Other** |  |  |
|  | 1648 |  |

|  |  | **Original GMM** | | | **Bootstrapped threshold (n=1118)** | | **Bootstrapped threshold (n=261)** | |
| --- | --- | --- | --- | --- | --- | --- | --- | --- |
| **ROI** | **mean n voxels** | **mean_lowGaussian_** | **sd_lowGaussian_** | **thresh** | **CI_low_** | **CI_high_** | **CI_low_** | **CI_high_** |
| precuneus_rh | 590 | 0.716 | 0.041 | 0.799 | 0.784 | 0.816 | 0.783 | 0.833 |
| precuneus_lh | 563 | 0.717 | 0.045 | 0.796 | 0.786 | 0.808 | 0.761 | 0.825 |
| inferiortemporal_lh | 677 | 0.696 | 0.053 | 0.784 | 0.765 | 0.798 | 0.749 | 0.846 |
| inferiortemporal_rh | 665 | 0.693 | 0.048 | 0.775 | 0.760 | 0.784 | 0.734 | 0.816 |
| inferiorparietal_lh | 700 | 0.681 | 0.049 | 0.758 | 0.746 | 0.771 | 0.697 | 0.861 |
| inferiorparietal_rh | 845 | 0.691 | 0.055 | 0.777 | 0.758 | 0.794 | 0.731 | 0.820 |
| superiorfrontal_rh | 1233 | 0.648 | 0.040 | 0.718 | 0.710 | 0.727 | 0.672 | 0.758 |
| superiorfrontal_lh | 1297 | 0.645 | 0.043 | 0.725 | 0.718 | 0.743 | 0.714 | 0.752 |
| middletemporal_lh | 655 | 0.656 | 0.052 | 0.742 | 0.727 | 0.753 | 0.700 | 0.768 |
| middletemporal_rh | 726 | 0.666 | 0.046 | 0.743 | 0.725 | 0.758 | 0.682 | 0.804 |
| rostralmiddlefrontal_lh | 867 | 0.665 | 0.044 | 0.753 | 0.743 | 0.766 | 0.734 | 0.778 |
| rostralmiddlefrontal_rh | 907 | 0.671 | 0.044 | 0.760 | 0.748 | 0.774 | 0.722 | 0.840 |
| medialorbitofrontal_rh | 344 | 0.681 | 0.044 | 0.756 | 0.746 | 0.769 | 0.723 | 0.801 |
| medialorbitofrontal_lh | 322 | 0.673 | 0.045 | 0.750 | 0.739 | 0.762 | 0.746 | 0.840 |
| rostralanteriorcingulate_rh | 114 | 0.722 | 0.054 | 0.802 | 0.786 | 0.826 | 0.793 | 0.859 |
| rostralanteriorcingulate_lh | 154 | 0.707 | 0.055 | 0.790 | 0.775 | 0.814 | 0.727 | 0.865 |
| caudalanteriorcingulate_rh | 114 | 0.714 | 0.056 | 0.825 | 0.813 | 0.841 | 0.769 | 0.854 |
| caudalanteriorcingulate_lh | 101 | 0.722 | 0.060 | 0.842 | 0.823 | 0.867 | 0.857 | 0.976 |
| posteriorcingulate_rh | 184 | 0.728 | 0.059 | 0.816 | 0.801 | 0.834 | 0.752 | 0.906 |
| posteriorcingulate_lh | 181 | 0.732 | 0.060 | 0.852 | 0.834 | 0.871 | 0.788 | 0.886 |
| lateralorbitofrontal_lh | 454 | 0.762 | 0.052 | 0.865 | 0.848 | 0.883 | 0.910 | 1.006 |
| lateralorbitofrontal_rh | 446 | 0.769 | 0.056 | 0.880 | 0.865 | 0.898 | 0.810 | 0.914 |
| caudalmiddlefrontal_lh | 358 | 0.698 | 0.049 | 0.774 | 0.756 | 0.789 | 0.724 | 0.799 |
| caudalmiddlefrontal_rh | 332 | 0.703 | 0.049 | 0.782 | 0.767 | 0.794 | 0.720 | 0.918 |
| superiorparietal_lh | 768 | 0.660 | 0.050 | 0.761 | 0.744 | 0.773 | 0.708 | 0.816 |
| superiorparietal_rh | 748 | 0.657 | 0.049 | 0.738 | 0.722 | 0.748 | 0.697 | 0.811 |
| isthmuscingulate_rh | 148 | 0.747 | 0.048 | 0.824 | 0.812 | 0.837 | 0.794 | 0.854 |
| isthmuscingulate_lh | 160 | 0.746 | 0.046 | 0.821 | 0.807 | 0.837 | 0.778 | 0.841 |
| parstriangularis_lh | 209 | 0.719 | 0.048 | 0.792 | 0.781 | 0.805 | 0.769 | 0.838 |
| parstriangularis_rh | 248 | 0.724 | 0.048 | 0.798 | 0.785 | 0.813 | 0.756 | 0.842 |
| parsopercularis_lh | 268 | 0.713 | 0.043 | 0.782 | 0.771 | 0.793 | 0.730 | 0.877 |
| parsopercularis_rh | 224 | 0.729 | 0.045 | 0.803 | 0.793 | 0.812 | 0.770 | 0.820 |
| parsorbitalis_lh | 140 | 0.683 | 0.059 | 0.801 | 0.771 | 0.831 | 0.654 | 0.975 |
| parsorbitalis_rh | 170 | 0.697 | 0.057 | 0.810 | 0.792 | 0.828 | 0.738 | 0.812 |
| insula_lh | 430 | 0.704 | 0.043 | 0.790 | 0.778 | 0.799 | 0.738 | 0.848 |
| insula_rh | 420 | 0.709 | 0.051 | 0.810 | 0.795 | 0.820 | 0.723 | 0.849 |
| bankssts_rh | 131 | 0.824 | 0.045 | 0.902 | 0.891 | 0.913 | 0.851 | 0.910 |
| bankssts_lh | 140 | 0.807 | 0.055 | 0.894 | 0.877 | 0.911 | 0.827 | 1.047 |
| supramarginal_lh | 660 | 0.685 | 0.043 | 0.771 | 0.758 | 0.785 | 0.740 | 0.805 |
| supramarginal_rh | 586 | 0.697 | 0.042 | 0.785 | 0.766 | 0.793 | 0.738 | 0.798 |
| superiortemporal_lh | 738 | 0.642 | 0.038 | 0.707 | 0.685 | 0.704 | 0.678 | 0.711 |
| superiortemporal_rh | 697 | 0.648 | 0.037 | 0.716 | 0.699 | 0.724 | 0.692 | 0.732 |

**Table S2. ROI distribution summary.** Summary data provide additional information about each ROI’s PIB DVR distribution at A4/LEARN baseline (n=1,118). The first column reports the mean number of voxels in each ROI. The next 3 columns report relevant output from each ROI’s fitted GMM: the mean of the lower gaussian, the standard deviation of the lower gaussian, and the GMM-derived threshold at the 50^th^ percentile for membership in either the lower or upper gaussian distribution. For most ROIs, this value fell between 2 and 3 standard deviations from the mean of the lower gaussian. Next, bootstrapped computation of the GMM (R=1000, with replacement) using the full A4/LEARN sample were used to estimate 95% confidence intervals around these thresholds. Finally, bootstrapping was repeated with a sample size limit of 261 subjects to align with our original EXT work in HABS, allowing for comparison in robustness of the ROI GMM thresholds due to tracer and camera rather than sample size.


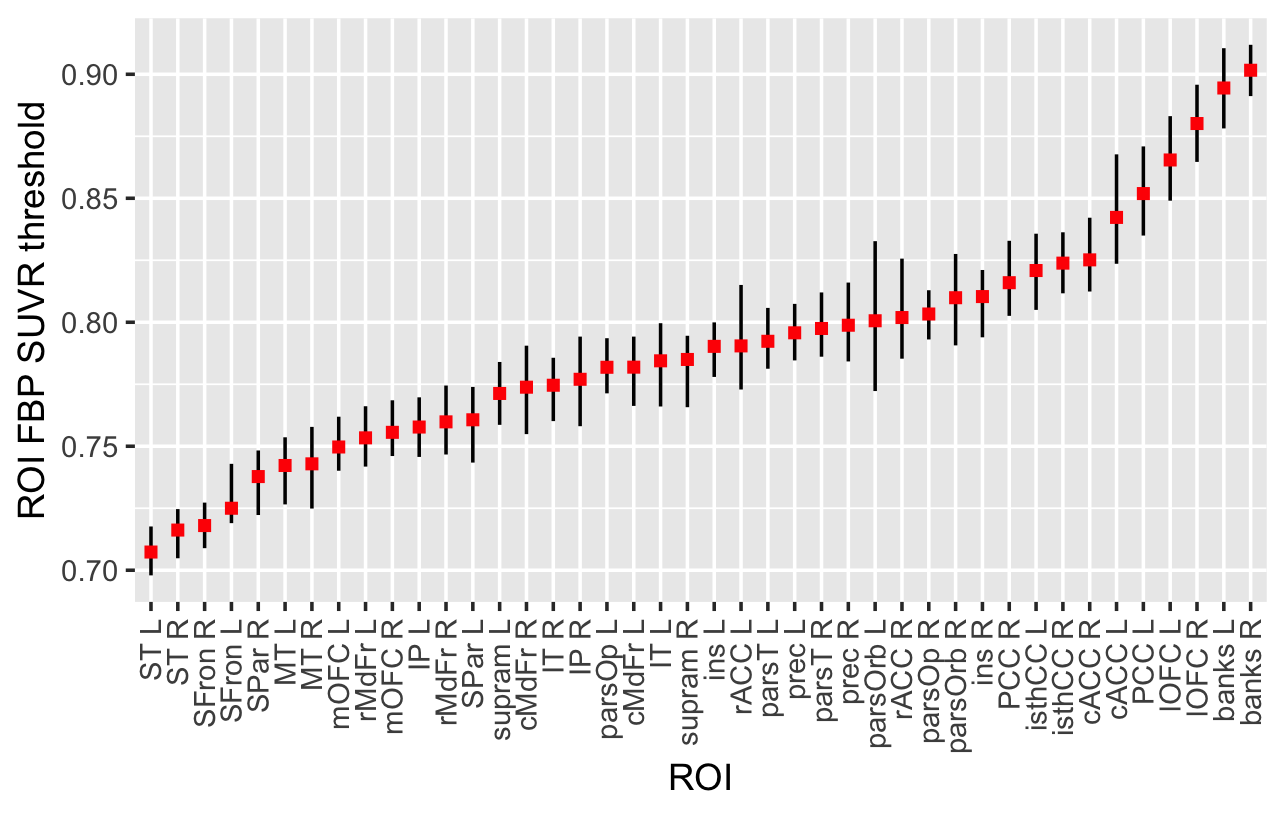


**Figure S1. Thresholds differ substantially by region.** The thresholds and 95% confidence intervals are shown for each ROI, generated using SUVRs calculated with the composite reference region.

**Analyses using a whole cerebellum reference region rather than the composite**


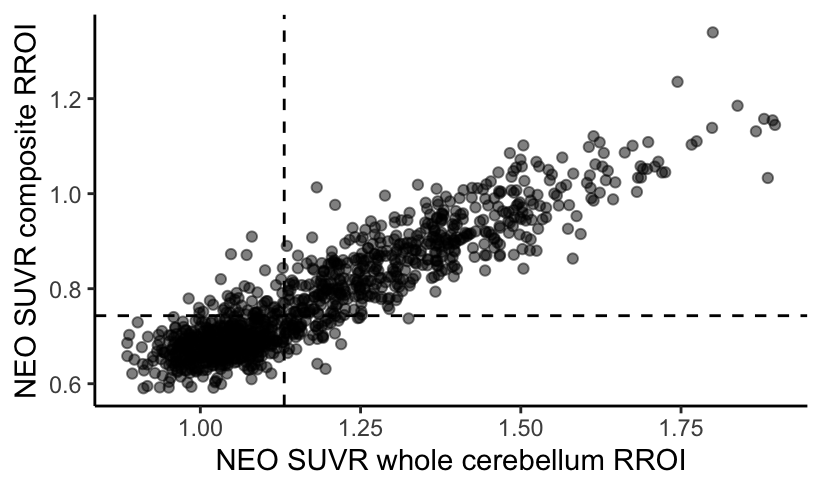


**Figure S2. SUVR computed with whole cerebellum reference region vs. composite.** A scatterplot displays baseline neocortical SUVR computed using a traditional whole cerebellum reference region (x-axis) versus the same SUVR computed using the composite reference region. Dashed lines indicate the GMM-derived positivity threshold for each version of the neocortical SUVR. Correspondence between these two versions was statistically high (R^2^=0.85), but since they are both intended to measure the same phenomenon 15% unshared variance is a notable difference.


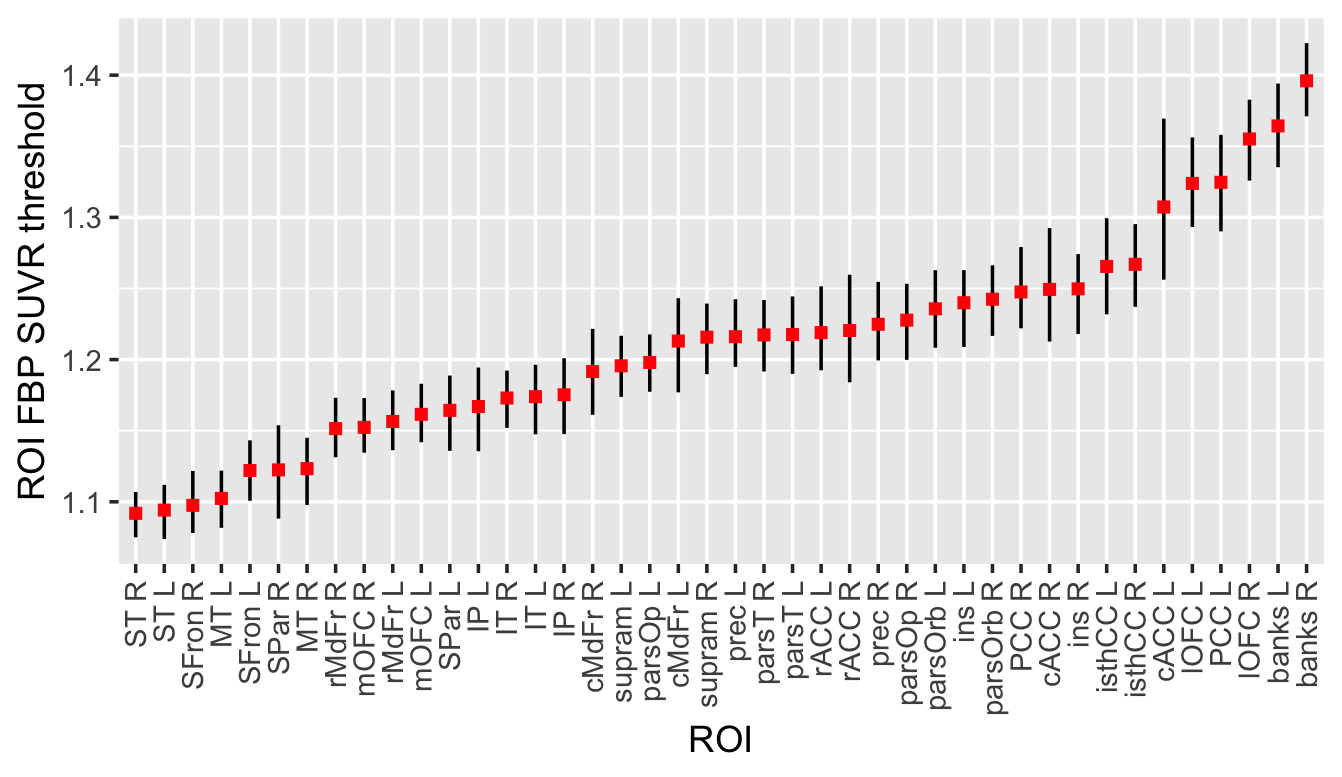


**Figure S3. Bootstrapped resampled thresholds and confidence intervals using whole cerebellum.** The same bootstrapping approach was applied to generate 95% confidence intervals around each ROI’s GMM threshold when using FBP SUVR that used whole cerebellum as the reference region. Relative to the results using the composite reference (Figure S1), there is more variability both within and between the ROI thresholds (note the shifted and expanded y-axis).


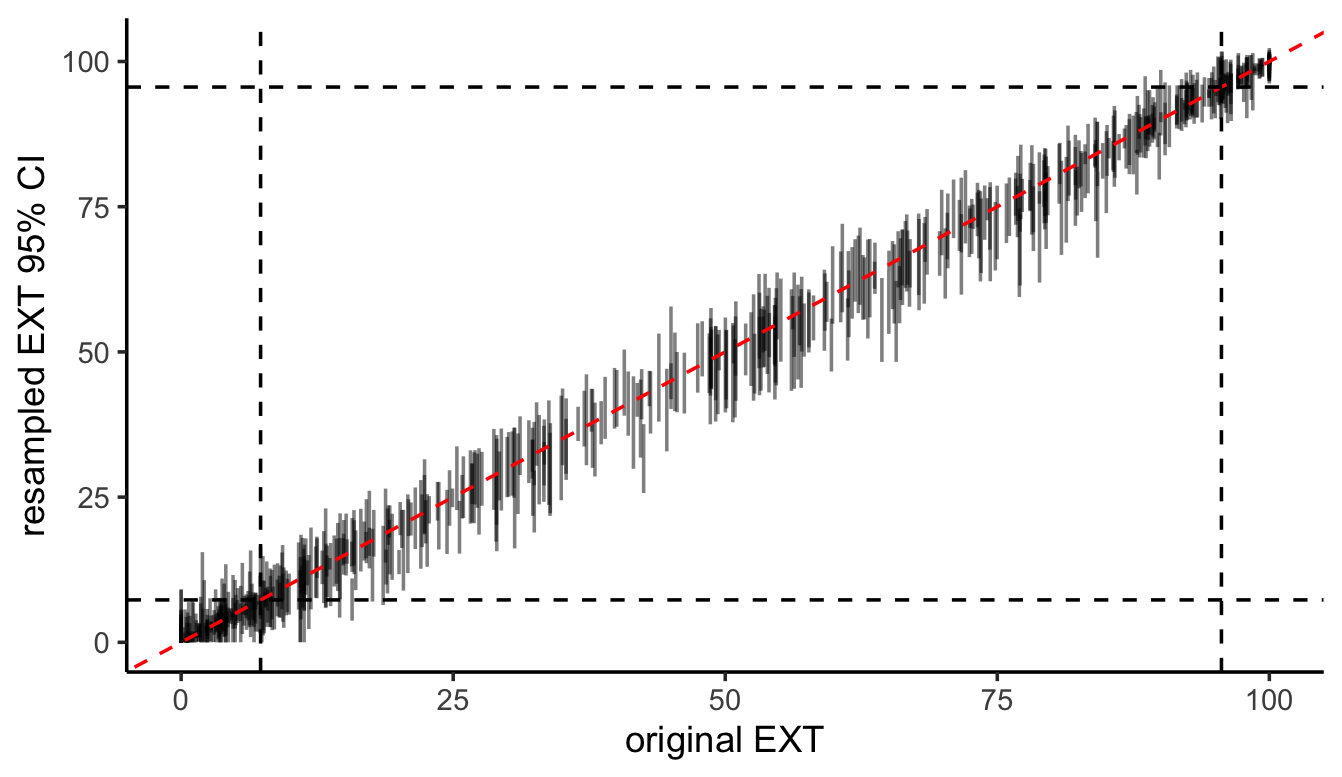


**Figure S4.** **Cross-sectional EXT reliability using** **the whole cerebellum reference region.** As in Figure 1B, bootstrapped samples of ROI GMM thresholds (see Figure S3) were used to generate 95% confidence intervals for each individual’s EXT value but with SUVR computed with whole cerebellum as the reference region instead of the composite. EXT values computed using a whole cerebellum reference region rather than the composite were more variable after resampling (M_D_= -0.33% (95% CI: -2.59, 2.04), p<.001) and more often resulted in changes in EXT staging (P=3.08%, χ^2^=461.78, p<.001).

**
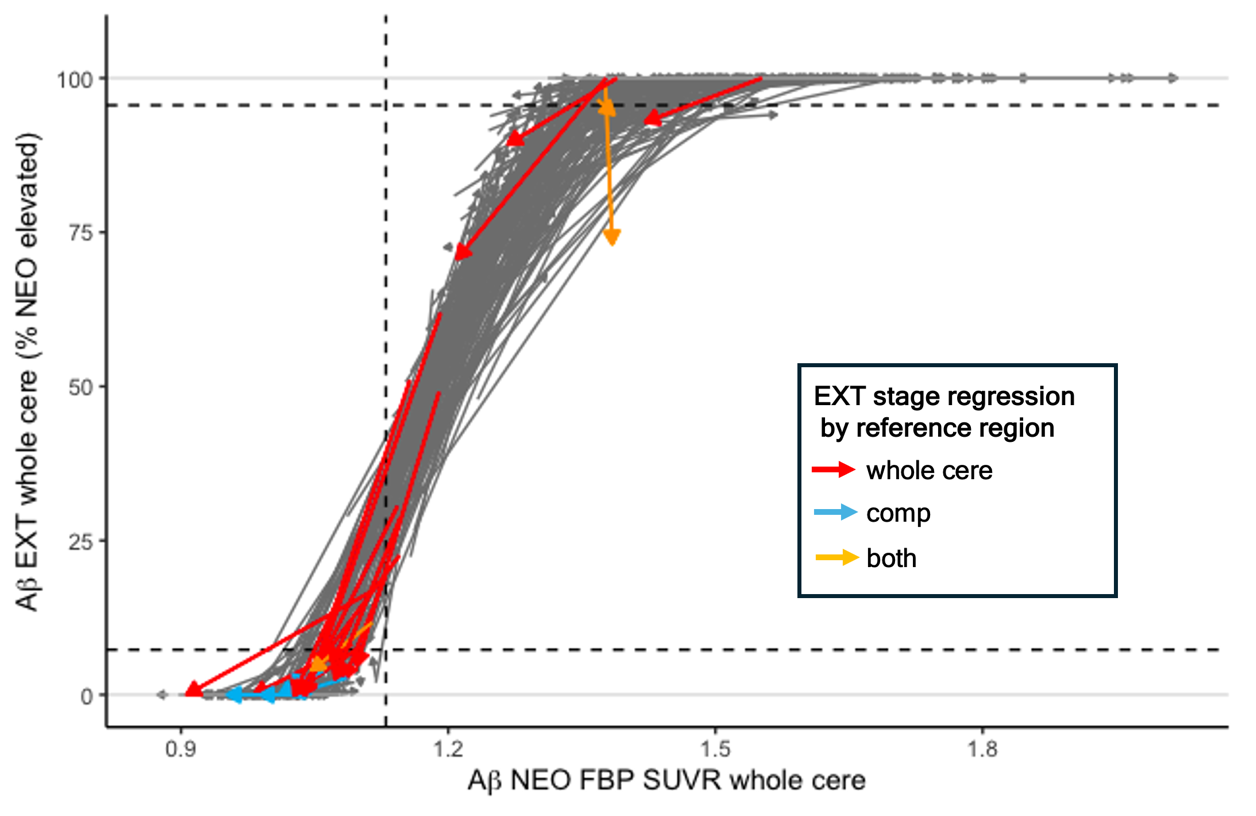
**

**Figure S5.** **Longitudinal EXT and SUVR more prone to declines and stage regressions when using the whole cerebellum reference region.** A spaghetti plot displays longitudinal Aβ changes measured with SUVR (x-axis) and EXT (y-axis) when both are measured with whole cerebellum as the reference region instead of the composite reference region. Individuals are colored to highlight the frequency of EXT stage regressions with the whole cerebellum vs. composite reference region. A total of 8 (1.2%) stage regressions were observed with the composite reference region compared to 18 (2.7%) stage regressions with whole cerebellum. Of these, 3 participants regressed with both reference regions, 5 regressed only when using the composite reference region and 15 regressed only when using whole cerebellum reference region. Furthermore, negative SUVR slopes occurred in 25% of subjects (n=168) versus 23.2% (n=156) with the composite. Negative EXT slopes occurred in 12.1% (n=81) of subjects when using whole cerebellum, while the composite yielded negative slopes only 9.2% (n=62) of the time. Overall, the use of the composite reference region reduced the frequency of longitudinally implausible declines in Aβ over time.


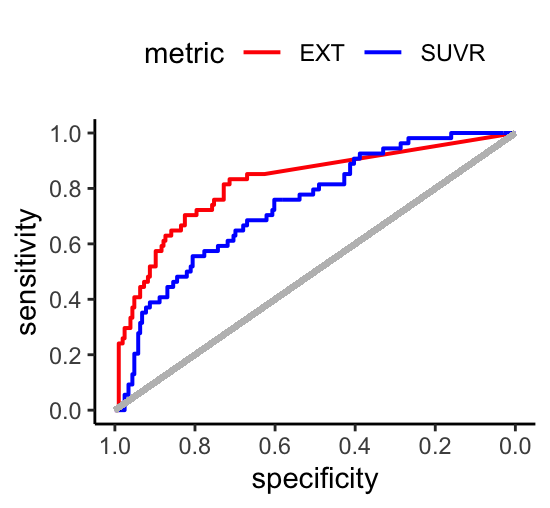


**Figure S6.** **Early detection of amyloidosis with EXT versus SUVR using the whole cerebellum reference region.** Using the whole cerebellum as the reference region for EXT and SUVR, receiver operator characteristic (ROC) analyses were conducted using baseline Aβ EXT (red) or SUVR (blue) to predict progression of SUVR- individuals to SUVR positivity over the 5.5-year follow-up this time RROI. Similar to the results for the composite reference region, EXT outperformed SUVR (AUC_EXT_=0.82 [CI: 0.75-0.88], AUC_SUVR_=0.74 [CI: 0.67-0.81]) but the results for both EXT and SUVR are weaker than observed with the composite reference region.

**
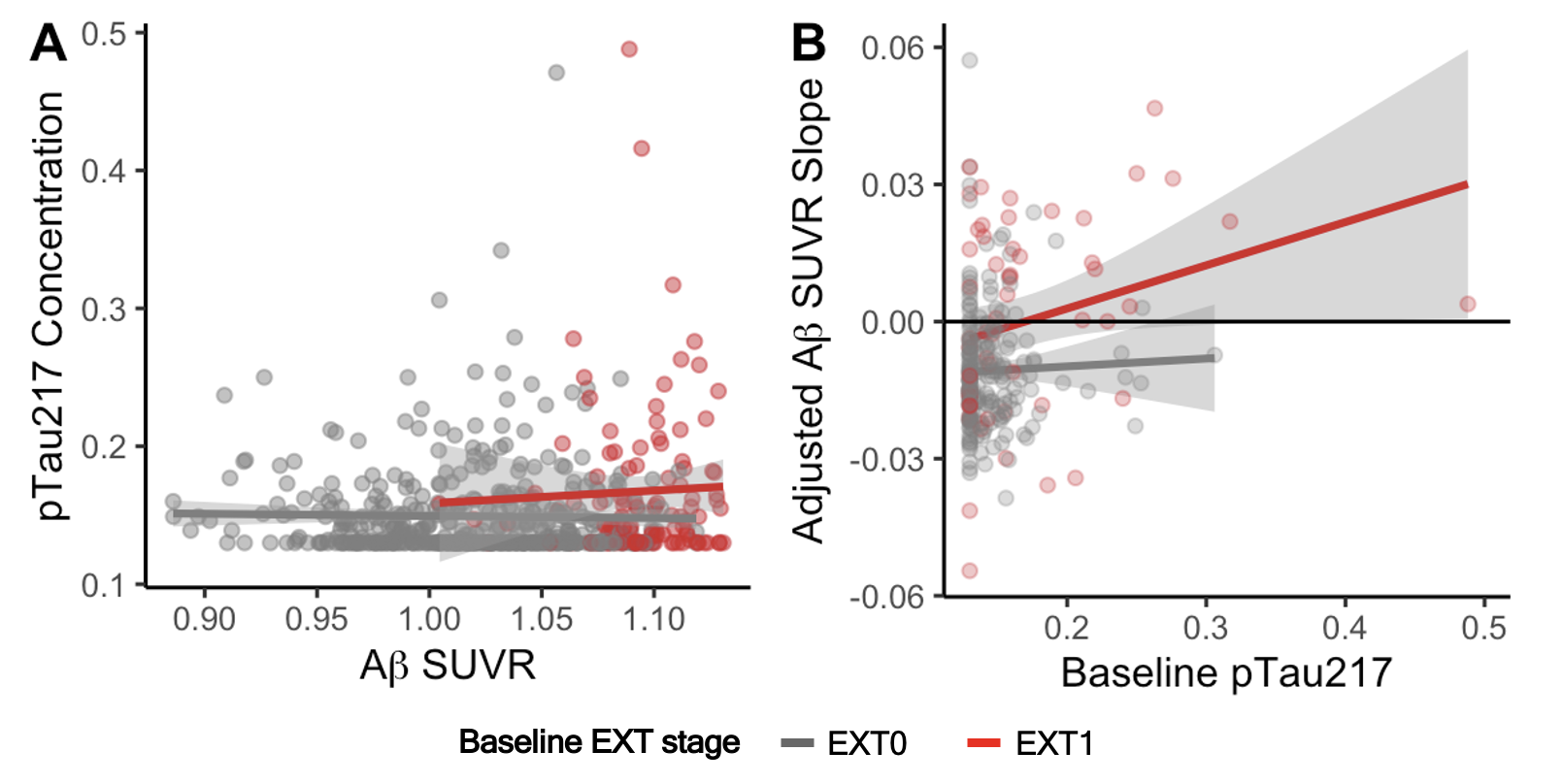
**

**Figure S7. Relationship of pTau217 concentration with Aβ PET in the SUVR- subsample using whole cerebellum reference (n_total_=549, n_LEARN_= 490, n_A4_=59).** A) Results using the whole cerebellum reference region for SUVR and EXT parallel those with the composite reference region, though they do not reach statistical significance. SUVR negativity was assigned to those with a baseline SUVR below a GMM threshold of 1.13 (approximately equivalent to 24CL). We observe that higher SUVR and higher pTau217 concentration go together within the SUVR- group, though they do not reach statical significance when using whole cerebellum (*β*=0.0728, *SE*=.048, *p*=.13). Introducing EXT group, we do see a significant difference between EXT1 (red, n_LEARN_= 76, n_A4_=30) and EXT0 (grey, n_LEARN_= 414, n_A4_= 29; *β*=0.0654, *SE*=.015, *p*<.001), however the within-group association between pTau217 and SUVR did not reach statistical significance (EXT1: *β*=0.0181, *SE*=.044, *p*=.682; EXT0: *β*= -0.0168, *SE*=.065, *p*=0.79). At these low levels of Aβ, the relationship of SUVR and pTau217 is not as strong as with the composite, likely because the use of the whole cerebellum reference region results in noisier, less reliable SUVR values and EXT classification. B) Higher baseline pTau217 concentration is associated with higher SUVR slope (*β*=0.0041, *SE*=.00036, *p*<.001) and differs by EXT group (*β*=0.0020, *SE*=.00087, *p*=.024). Aβ SUVR increase over time is predicted by baseline pTau217 for both groups here (EXT1: *β*=0.00645, *SE*=.00099, *p*<.001; EXT0: *β*=0.00105, *SE*=.00044, *p*=.018). However, the individuals who demonstrate associations between increasing SUVR over time and higher baseline pTau217 tend to be in the EXT1 group, making the magnitude of the effect much larger in EXT1. This further reinforces that the pattern of results is similar to those using a composite reference.


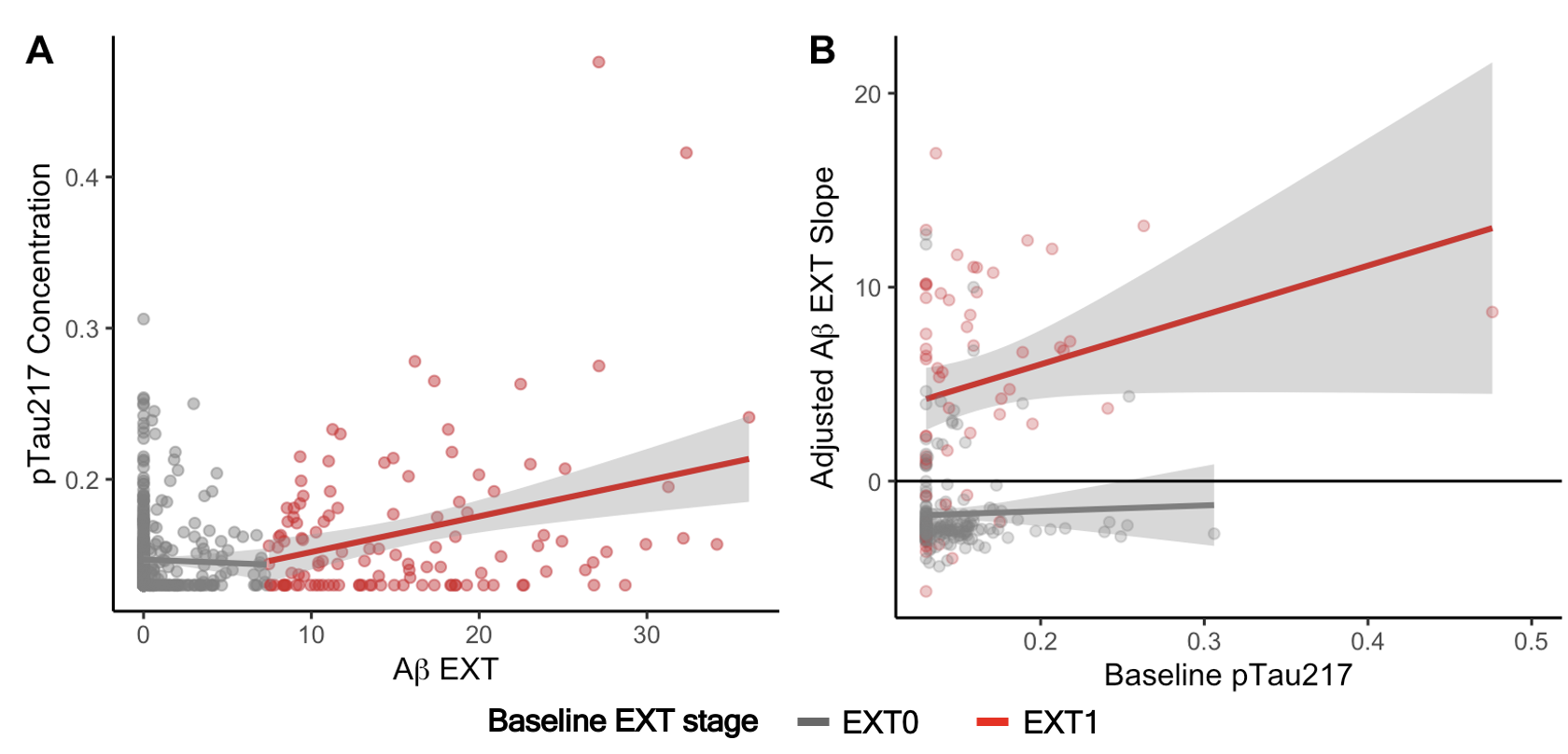


**Figure S8. Baseline Aβ spatial extent (EXT) and plasma pTau217 in SUVR- participants.** A) A scatterplot depicts the cross-sectional association between Aβ EXT and plasma pTau217 concentration at baseline, colored by EXT stage. Solid lines represent model-estimated associations within each EXT stage, with shaded areas indicating 95% confidence intervals. Higher Aβ EXT was associated with higher plasma pTau217 only among individuals classified as EXT1. B) The longitudinal association is shown between baseline plasma pTau217 and change in Aβ EXT (annualized Aβ EXT slope, adjusted for age, sex, APOE and education), and colored by baseline EXT stage. Among individuals classified as EXT1 at baseline, higher pTau217 was associated with greater subsequent Aβ spread, whereas no significant association was observed in EXT0. These findings support that elevated ptau217 concentration below traditional global SUVR thresholds can be taken as a marker of Aβ accumulation if corroborated with Aβ EXT.

| **Outcome** |  | **Independent Models** | | | | | | **Combined Models** | | | |
| --- | --- | --- | --- | --- | --- | --- | --- | --- | --- | --- | --- |
|  | **Sample** | **Metric** | **β** | **SE** | **p** | **η^2^** | **AIC** | **β** | **SE** | **p** | **AIC** |
| **PACC** | **All** | SUVR | -0.202 | 0.019 | <.001 | **0.10** | **44491** | -0.222 | 0.041 | <.001 | 44493 |
|  |  | EXT | -0.181 | 0.019 | <.001 | 0.08 | 44519 | 0.021 | 0.032 | 0.654 |  |
|  | **EXT0\|1** | SUVR | -0.128 | 0.041 | 0.001 | 0.016 | 29088 | 0.0237 | 0.088 | 0.788 | 29086 |
|  |  | EXT | -0.119 | 0.032 | <.001 | **0.022** | **29083** | -0.136 | 0.070 | 0.053 |  |
|  | **EXT<50%** | SUVR | -0.074 | 0.054 | 0.172 | 0.004 | 24826 | 0.036 | 0.089 | 0.688 | 24823 |
|  |  | EXT | -0.119 | 0.058 | 0.042 | **0.008** | **24822** | -0.151 | 0.096 | 0.119 |  |
| **MTL TAU** | **All** | SUVR | 0.010 | 0.003 | <.001 | 0.06 | **-783** | 0.001 | 0.006 | 0.915 | -782 |
|  |  | EXT | 0.011 | 0.003 | <.001 | **0.07** | -771 | 0.011 | 0.005 | 0.102 |  |
|  | **EXT0\|1** | SUVR | 0.015 | 0.006 | 0.020 | 0.048 | **-671** | 0.010 | 0.016 | 0.560 | -668 |
|  |  | EXT | 0.011 | 0.005 | 0.022 | **0.050** | -667 | 0.004 | 0.012 | 0.7161 |  |
|  | **EXT<50%** | SUVR | 0.013 | 0.009 | 0.155 | 0.027 | **-508** | -0.004 | 0.017 | 0.835 | -505 |
|  |  | EXT | 0.014 | 0.008 | 0.072 | **0.047** | -504 | 0.017 | 0.015 | 0.281 |  |
| **nTEMP TAU** | **All** | SUVR | 0.028 | 0.004 | <.001 | 0.17 | -1020 | 0.014 | 0.009 | 0.124 | **-1022** |
|  |  | EXT | 0.027 | 0.004 | <.001 | 0.17 | -1013 | 0.015 | 0.009 | 0.111 |  |
|  | **EXT0\|1** | SUVR | 0.021 | 0.005 | <.001 | 0.14 | -967 | -0.007 | 0.013 | 0.591 | -970 |
|  |  | EXT | 0.017 | 0.004 | <.001 | **0.19** | **-971** | 0.022 | 0.009 | 0.023 |  |
|  | **EXT<50%** | SUVR | 0.014 | 0.006 | 0.017 | 0.07 | -760 | -0.005 | 0.011 | 0.627 | -761 |
|  |  | EXT | 0.016 | 0.005 | 0.002 | **0.13** | **-763** | 0.021 | 0.010 | 0.044 |  |

**Table S3.** **Linear mixed-effects model results examining the predictive power of baseline amyloid burden (SUVR) and spatial extent (EXT) on cognitive decline (PACC) and tau accumulation (MTL and nTEMP) with whole cerebellum RROI yield weaker differentiation between EXT and SUVR**. Similar to the analyses with composite RROI-derived values, three models were performed for each outcome. First, independent models were run to test the association between each Aβ metric at baseline on each outcome over time (Model 1: time*SUVR, Model 2: time*EXT). EXT and SUVR were standardized to allow for direct comparison of the β estimates and standard error between models. Effect sizes (partial η²) and Akaike Information Criterion (AIC) are reported to provide estimates of EXT and SUVR’s effect on each outcome and overall model fit. Next, we conducted a combined model including the time*EXT and time*SUVR terms in the same model to see which measure was a better fit to the data. AIC for the combined model is also reported. Analyses were repeated within different subsamples representing different stages of amyloidosis, decreasing Aβ load from the full sample (All) to EXT0|1 to EXT<50%. The overall pattern of findings is similar to those seen with the composite reference region, but effect sizes are weaker. Furthermore, for some outcomes/subsamples, the overall model fit is better with SUVR but the effect size for the time*EXT is greater than the time*SUVR effect size. This resulted from poorer prediction of change over time with the whole cerebellum version of the Aβ metrics, such that the main effect of SUVR was the strongest Aβ effect on tau/cognition.

**Section S2. Exploring tau proliferation and cognitive decline in SUVR-based groups.** Three SUVR groups (SUVR−/SUVR+/SUVR++) were derived using thresholds corresponding to the intersection points of the EXT1 and EXT2 thresholds with the logistic growth curve shown in Figure 1A. These SUVR thresholds (0.705 and 0.870) were selected to approximate the staging defined by EXT and resulted in assignment discordance primarily within the intermediate range (EXT1 and SUVR+) as summarized in Table S4. However, as demonstrated by the ROC in Section 3.4, adoption of a low SUVR+ thresholds that best approximate EXT’s ability to detect early focal Aβ has the drawback of adding potential false positives to the SUVR+ group that are EXT0.

|  | Full sample | | | Tau subsample | | |
| --- | --- | --- | --- | --- | --- | --- |
|  | SUVR- | SUVR + | SUVR ++ | SUVR- | SUVR + | SUVR ++ |
| EXT0 | 404 | 48 | 0 | 57 | 6 | 0 |
| EXT1 | 25 | 357 | 38 | 3 | 95 | 15 |
| EXT2 | 0 | 14 | 232 | 0 | 7 | 63 |

**Table S4.** **Concordance between EXT stages and SUVR-based groups using the LGM derived thresholds in the full cohort and tau subsample.**

Using these SUVR stages to predict tau and cognition over time (Figure S9), we observed a similar overall pattern but with slightly weaker differences between Aβ- stages (SUVR-, EXT0) and the Aβ+ stages (SUVR+/++, EXT1/2). Results in the SUVR- group closely mirrored the EXT0 stage, with small increases over time in MTL tau (SUVR-: β=0.020, SE=.006, p=.001; EXT0: β=0.018, SE=.006, p=.003), no change in nTEMP tau (SUVR-: β=0.012, p=.155; EXT0: β=0.012, p=.131), and a practice effect on PACC (SUVR-: β=0.146, p<.001; EXT0: β=0.162, SE=.034 p<.001). Detection of an increase in tau change over time from the SUVR- to SUVR+ stage were similar but very slightly weaker than between EXT0 and EXT1 for both MTL (SUVR+: β=0.014, SE=.007, p=.035, EXT1: β=0.015, SE=.007, p=.018) and nTEMP (β=0.021, SE=.009, p=.018, EXT1: β=0.022, SE=.009, p=.015). However, for the PACC the significant difference between EXT1 and EXT0 (β= -0.126, SE=.043, p=.003, Figure 3C) was mitigated to a trend when comparing the SUVR+ and SUVR- stages (β= -0.083, SE=.043, p=.057, Figure S9C).

**
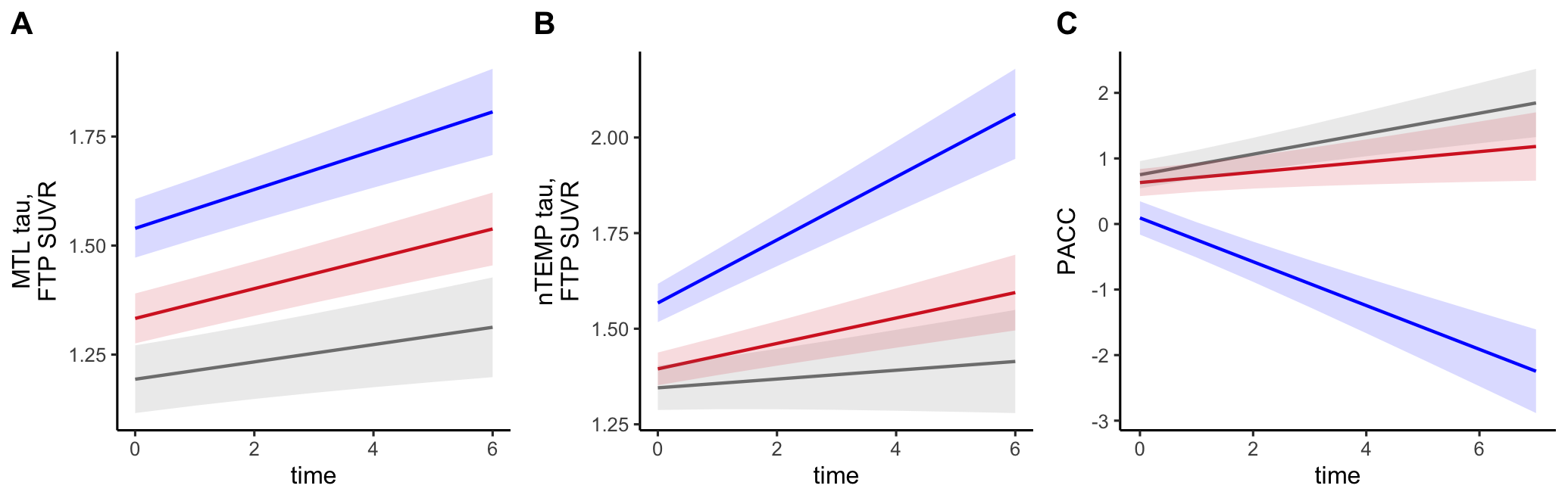
**

SUVR++

SUVR-

SUVR+

**Baseline SUVR stage**

**Figure S9.** **Relationships of baseline EXT-based SUVR groups with tau and cognition change.** Estimated marginal means from the linear mixed effect models of the baseline SUVR stage*time effect on A) MTL tau, B) nTEMP tau and C) PACC are shown for the average participant (71.8 year-old female with 16.0 years of education). Participants were categorized into SUVR- (gray), SUVR+ (red), and SUVR++ (blue) groups based on thresholds of 0.705 and 0.870 derived from EXT logistic growth modeling. Consistent with EXT-based staging, SUVR- individuals demonstrated modest MTL tau increase, no significant neocortical tau change, and a practice effect on PACC. SUVR+ individuals exhibited accelerated MTL and emerging nTEMP tau accumulation with attenuation of the practice effect. SUVR++ individuals showed the greatest longitudinal tau proliferation in both MTL and nTEMP regions and marked cognitive decline over time, demonstrating a graded, stage-dependent pattern of biological and cognitive worsening.

At the most advanced stage (SUVR++/EXT2), both staging approaches again identified the same overall pattern of marked worsening relative to the Aβ- stage (SUVR-/EXT0) but with weaker estimates using SUVR++ than with EXT2. Tau increased over time in the MTL (β=0.025, SE=.007 ,p<.001; EXT2: β=0.032, SE=.007, p<.006) and nTEMP (β=0.071, SE=.010, p<.001, β=0.078, SE=.010, p<.001) while PACC declined (SUVR2: β= -0.489, SE=.050, p<.001; EXT2: β= -0.525, SE=.051, p<.001). Overall, while both staging schemes capture the same stepwise biological pattern, EXT more selectively identifies individuals with early meaningful Aβ spread, reducing misclassification and sharpening prediction of downstream tau proliferation and cognitive decline.

We also generated a second SUVR staging schema (Table S5), keeping the same SUVR++ threshold but switching to the traditional GMM-derived cutoff SUVR+ cutoff (0.743) to reduce inclusion of SUVR+/EXT0 false positives.

|  | Full sample | | | Tau subsample | | |
| --- | --- | --- | --- | --- | --- | --- |
|  | SUVR- | SUVR + | SUVR ++ | SUVR- | SUVR + | SUVR ++ |
| EXT0 | 452 | 0 | 0 | 63 | 0 | 0 |
| EXT1 | 122 | 243 | 55 | 27 | 68 | 18 |
| EXT2 | 0 | 7 | 239 | 0 | 3 | 67 |

**Table S5.** **Concordance between EXT stages and SUVR-based groups using the GMM derived threshold in the full cohort and tau subsample.**

However, this also results in a SUVR+ stage that starts later in the Aβ continuum than the EXT1 stage. Unsurprisingly, estimates of tau change in this later SUVR+ group are slightly higher (MTL: β=0.019, SE=0.007, p<.001; nTEMP: β=0.025, SE=0.009, p=.006) than for EXT1 (MTL: β=0.015, nTEMP: β=0.022) . However, cognitive change remained attenuated in SUVR1 (PACC: β=0.032, SE=0.048, p=.012) relative to EXT1 (β=−0.126), suggesting that spatially defined EXT staging continues to provide sharper discrimination of clinically meaningful early Aβ spread.
